# Systematic investigation of CO_2_ : NH_3_ ice mixtures using mid-IR and VUV spectroscopy – part 2: electron irradiation and thermal processing†

**DOI:** 10.1039/d1ra05600j

**Published:** 2021-10-07

**Authors:** Rachel L. James, Sergio Ioppolo, Søren V. Hoffmann, Nykola C. Jones, Nigel J. Mason, Anita Dawes

**Affiliations:** School of Physical Sciences, The Open University, Walton Hall Milton Keynes UK Rachel.James1@open.ac.uk; School of Electronic Engineering and Computer Science, Queen Mary University of London Mile End Road London UK; ISA, Department of Physics and Astronomy, Aarhus University Ny Munkegade 120 DK-8000 Aarhus C Denmark; School of Physical Sciences, University of Kent Canterbury Kent UK

## Abstract

Many experimental parameters determine the chemical and physical properties of interstellar ice analogues, each of which may influence the molecular synthesis that occurs in such ices. In part 1, James *et al.*, *RSC Adv.*, 2020, **10**, 37517, we demonstrated the effects that the stoichiometric mixing ratio had on the chemical and physical properties of CO_2_ : NH_3_ mixtures and the impact on molecular synthesis induced by thermal processing. Here, in part 2, we extend this to include 1 keV electron irradiation at 20 K of several stoichiometric mixing ratios of CO_2_ : NH_3_ ices followed by thermal processing. We demonstrate that not all stoichiometric mixing ratios of CO_2_ : NH_3_ ice form the same products. Not only did the 4 : 1 ratio form a different residue after thermal processing, but O_3_ was observed after electron irradiation at 20 K, which was not observed in the other ratios. For the other ratios, the residue formed from a thermal reaction similar to the work shown in Part 1. However, conversion of ammonium carbamate to carbamic acid was hindered due to electron irradiation at 20 K. Our results demonstrate the need to systematically investigate stoichiometric mixing ratios to better characterise the chemical and physical properties of interstellar ice analogues to further our understanding of the routes of molecular synthesis under different astrochemical conditions.

## Abstract 2

The stoichiometric mixing ratio of CO_2_ : NH_3_ ice mixtures determines the electron irradiation products at 20 K and the composition of residue material formed after thermal processing.

## Graphical abstract

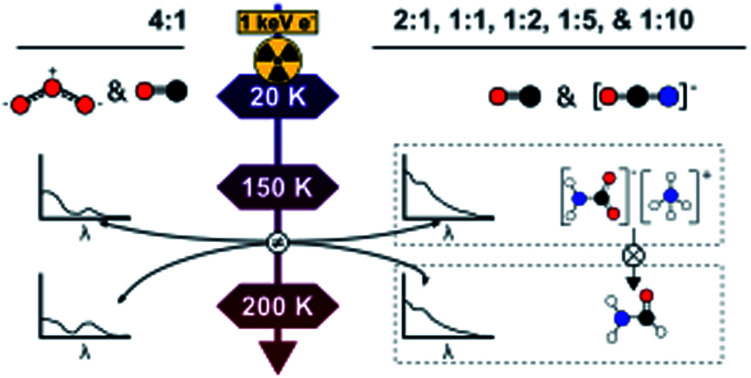

## 1 Introduction

Interstellar ice analogue experiments play an important role in understanding molecular synthesis in the interstellar medium (ISM), and systematic investigations into the experimental parameters can provide a wealth of information. In James *et al.*,^1^ henceforth referred to as RJ20, we demonstrated the impact that one discrete experimental parameter, the stoichiometric mixing ratio, has on both the chemical and physical properties of CO_2_ : NH_3_ ice mixtures from deposition at 20 K and throughout thermal processing. For example, the NH_3_-rich CO_2_ : NH_3_ mixtures (*e.g.* 1 : 3, 1 : 10) formed higher amounts of residue material where more NH_3_ crystallite grain boundaries existed, suggesting that structural diffusion of reactants may be linked to enhanced reactivity of this system.

In this follow-up paper, we extend our study of the discrete experimental parameter of stoichiometric mixing ratios of CO_2_ : NH_3_ ices to include 1 keV electron irradiation at 20 K followed by thermal processing. Non-thermal processing due to electrons is thought to occur in the ISM due to the interaction of cosmic rays with solids releasing secondary electrons.^2^ Electron irradiation of CO_2_ : NH_3_ ice analogues has been previously studied^3,4^ as well as other methods of non-thermal processing such as UV photons^5^ and ions.^6^ These previous studies are summarised in Table 1. Ammonium carbamate ([NH_4_][H_2_NCO_2_]) and carbamic acid (H_2_NCOOH) were reported as UV processing products at 10 K, ammonium carbamate was reported as a product for 9–20 eV electron irradiation at 10 K and carbamic acid as a product for 144 keV S^9+^ ion processing at 16 K. We note that ammonium carbamate, or both ammonium carbamate and carbamic acid, were identified as the thermal products in previous non-irradiated studies.^1,3,5–12^ In addition to ammonium carbamate and/or carbamic acid CO,^4,5^ OCN^−^,^4,5^ N_2_O (ref. 6) and ammonium formate^5,6^ were reported. In the previous studies of non-thermal processing of CO_2_ : NH_3_ ices summarised in Table 1 only two different stoichiometric mixing ratios were investigated, a 1 : 1 ratio and a 0.75 : 1 ratio. As such, this paper presents the first study dedicated to investigating the non-thermal processing of different stoichiometric mixing ratios of CO_2_ : NH_3_ ices.

**Table 1:** Summary of the results of previous studies conducted on the non-thermal processing of CO_2_ : NH_3_ ice mixtures at low temperatures (10–30 K). Deposition temperature (*T*_d_), product formation temperature (*T*_f_)

Reference	CO_2_ : NH_3_ ratio	*T*_d_ (K)	Processing type	Processing fluence (particles per cm^2^)	Main products & *T*_f_ (K)
Bossa *et al.*^5^	1 : 1^a^	10	VUV photons	4.3 × 10^19^	AC (10 K, 110–230 K), AF (10 K), CA (10 K, 110–230 K), CO (10 K) & OCN^−^ (10 K)
Bertin *et al.*^3^	1 : 1^b^	10	9–20 eV electrons + thermal	Few 10^14^	AC (10 & 140 K), CA (140 K)
Jheeta *et al.*^4^	1 : 1^c^	30	1 keV electrons + thermal	4.6 × 10^17^	CO (30 K), NH^+^_4_ (30 K), OCN^−^ (30 K) & AC (254 K)
Lv *et al.*^6^	1 : 1, 0.75 : 1^b^	16	144 keV S^9+^ ions	(1.3 & 6.8) × 10^14^	AF (16 K), CA (16 K), N_2_O (16 K) & OCN^−^ (16 K)

a Method of determining the ratio not specified.

b Ratios derived from partial pressures of the mixture in the gas line.

c Ratios derived from column density, AC = ammonium carbamate, CA = carbamic acid.

Similar to RJ20, in this paper, we present combined mid-IR and vacuum-ultraviolet (VUV) studies of CO_2_ : NH_3_ ice mixtures. For the studies summarised in Table 1, *in situ* mid-IR spectroscopy was used to study the evolution of the CO_2_ : NH_3_ ices. For some studies, mass spectrometry^5^ and high-resolution low energy electron loss spectroscopy^3^ were also used. VUV spectroscopy not only provides an additional technique to study the formation of products, but variations in Rayleigh scattering tails in CO_2_ : NH_3_ ices observed in VUV spectra in RJ20 can provide insight into the physical changes occurring with the ice analogues due to initial stoichiometric mixing ratios.

In this paper, we present the results of CO_2_ : NH_3_ mixtures deposited at 20 K and then irradiated with 1 keV electrons at 20 K, and subsequent thermal processing. Henceforth ices processed in this way are referred to as e-irradiated. Mid-IR spectra of CO_2_ : NH_3_ mixtures with differing ratios of 4 : 1, 2 : 1, 1 : 1, 1 : 2, 1 : 5 & 1 : 10 are complemented by a VUV spectroscopic study of mixtures with ratios of 4 : 1, 2 : 1 & 1 : 3. The results presented in RJ20, which were also thermally processed, but not irradiated with electrons, are used as a set of control experiments to make direct comparisons and, therefore, investigate the effect of electron irradiation on the chemical and physical properties of the ice mixtures. The astrophysical implications of this study combined with that of RJ20 will be presented in a further publication.

## 2 Experimental

Both the mid-IR and VUV experiments were performed using The Open University Portable Astrochemistry Chamber (PAC). This was the same experimental system used in RJ20, using a Nicolet Nexus 670 FTIR spectrometer with an external MCT detector for mid-IR measurements and the AU-UV beamline on ASTRID2 (Aarhus University, Denmark) to record the VUV spectra. A more detailed description can be found in Section 2 of RJ20 and Section S1.1 of the ESI for RJ20. Physical vapour deposition of the ices was conducted at a base pressure of low 10^−9^ mbar and a base temperature of 20 K. CO_2_ (99.8%, BOC) and NH_3_ (99.96%, ARGO International Ltd) were premixed in the gas line prior to deposition and deposited onto a cooled substrate (mid-IR: ZnSe, Crystran; VUV: MgF_2_, Crystran). After deposition, the ice samples were irradiated with 1 keV electrons using a Kimball Physics FRA-2X1-5549 electron gun with a current of 10 μA. Electron irradiation was conducted at set intervals, cumulating in a total electron irradiation time of 30 min or a total fluence of 3.37 × 10^16^ e^−^ cm^−2^, and spectroscopic measurements were taken after each interval. The acquisition time for each mid-IR spectrum was approximately 2 min. After electron irradiation, the ices in the mid-IR study were allowed to rest for ∼1 h. For the VUV spectrum, the acquisition time is dependent on the step size used: for spectra below 110 K, this corresponded to ∼1 h, and for temperatures at or above 120 K, this corresponded to ∼10 min. The ice samples were then thermally processed to set temperatures and held at this temperature while spectroscopic measurements were taken.

The film thickness and ratios of the CO_2_ : NH_3_ mixtures were calculated in the same way as described in the ESI of RJ20. Deposition rates were between 0.8–1.9 nm s^−1^ for both mid-IR and VUV spectroscopic studies. The average film thickness for the mid-IR study was 437 nm. Thinner films for the VUV measurements were required to prevent saturation of the absorption peaks, and the average film thickness was 204 nm for the VUV study. The spectra of the mixtures were normalised to a specific thickness when compared, 400 nm for the mid-IR samples and 200 nm for the VUV samples, and indicated in the figure captions. The individual sample thickness and normalisation factors are given in Table S1 in the ESI.†

All mid-IR and VUV spectra are freely available on the Open Research Data Online (ORDO) Repository.^13^

### 2.1 Penetration depth of 1 keV electrons

To negate any substrate effects which may occur during electron irradiation the ice samples were grown to a thickness larger than the electron penetration depth. The penetration depths of the 1 keV electrons within the ice samples were estimated using the CASINO (monte CArlo SImulation of electroN trajectory in sOlids) program.^14^ The CASINO program requires inputs of the electron energy, the angle of the electron beam with respect to the sample, the composition, and density of the sample. A weighted density was used for the CO_2_ : NH_3_ mixtures which was calculated using the density of pure CO_2_ (1.11 g cm^−3^) and pure NH_3_ (0.74 g cm^−3^)^15^ and specific values are given in Table S2 of the ESI.† The estimated average penetration depth of the electrons was 65 nm, well below the sample thicknesses.

## 3 Mid-IR results

### 3.1 Deposition at 20 K

The following CO_2_ : NH_3_ mixtures were deposited at 20 K: 4 : 1, 2 : 1, 1 : 1, 1 : 2, 1 : 5 & 1 : 10. These ratios are similar to the ratios used in the mid-IR study of RJ20 (3 : 1, 2 : 1, 1 : 1, 1 : 3 & 1 : 10), with slight differences arising due to practical difficulties during gas mixing prior to deposition. In this study, the 4 : 1 ratio is comparable to the RJ20 3 : 1 ratio, and the 1 : 2 & 1 : 5 ratios are comparable to the RJ20 1 : 3 ratio.

Deposition spectra are shown in Section 2.1, Fig. S2.1 of the ESI,† and band assignments and positions are given in Table S3 of the ESI.† The same general trends in the mid-IR spectra were observed for the ratios used in this study and for the ratios used RJ20. In brief, the stoichiometric mixing ratio affected the CO_2_ bonding environment. For the NH_3_-rich 1 : 10 mixture, CO_2_ was essentially a defect within the NH_3_ ice and existed in the form of isolated CO_2_ molecules. For the NH_3_-rich 1 : 5 mixture CO_2_ complexed to NH_3_ to form a CO_2_ : NH_3_ molecular complex as well as existing as isolated CO_2_ molecules. Within the other ratios (4 : 1, 2 : 1, 1 : 1 & 1 : 2) CO_2_ was in the form of CO_2_ dimers, CO_2_ : NH_3_ molecular complexes and isolated CO_2_. For a detailed characterisation of the CO_2_ : NH_3_ mixtures deposited at 20 K, see Section 3.1 of RJ20.

### 3.2 Electron irradiation at 20 K

**Fig. 1:**
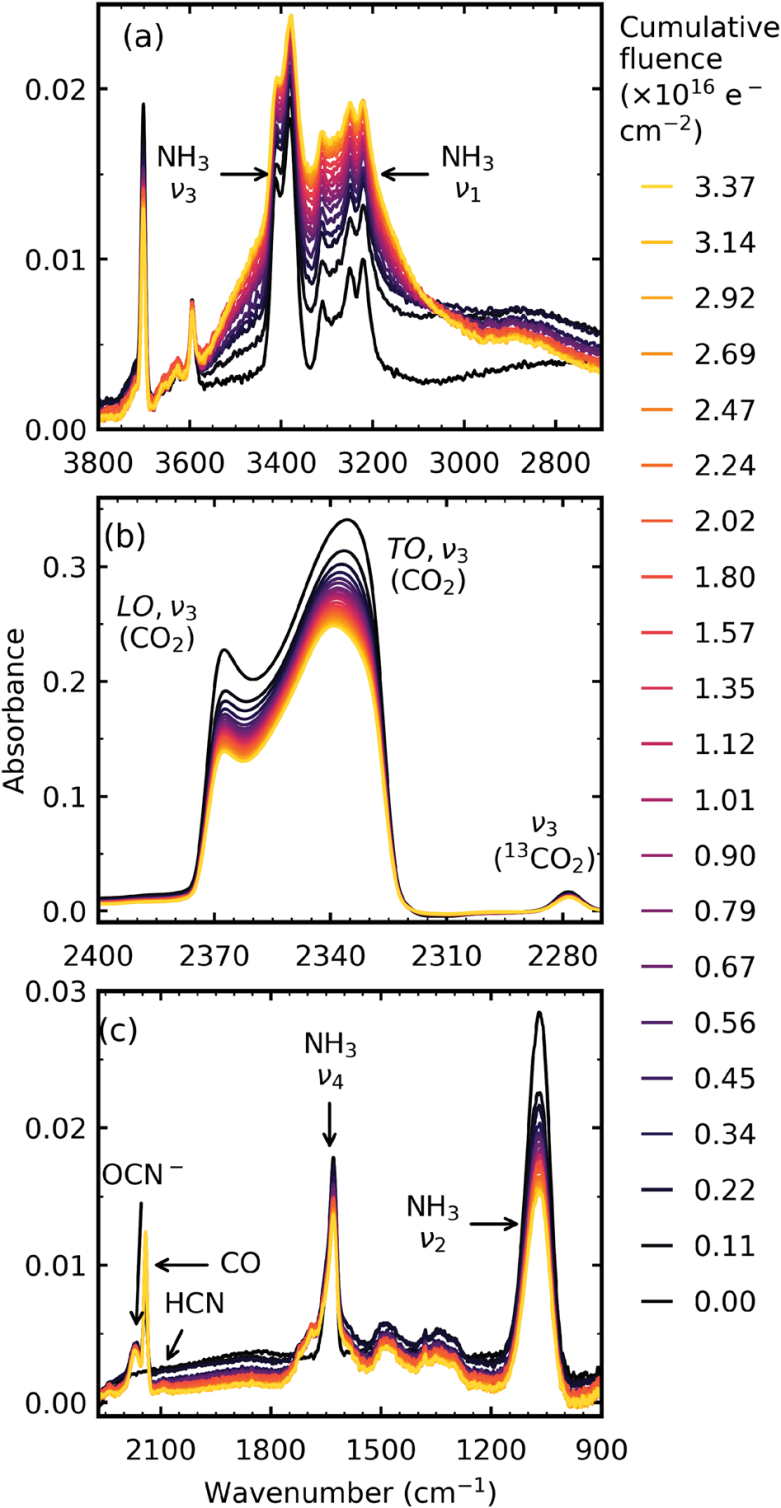
Example mid-IR spectra of a CO_2_ : NH_3_ mixture in a 1 : 1 ratio irradiated with 1 keV electrons at 20 K to a total fluence of 3.37 × 10^16^ e^−^ cm^−2^. See Section S2.2 of the ESI† for the mid-IR spectra of the 4 : 1, 2 : 1, 1 : 2, 1 : 5 & 1 : 10 ratios. (a) O–H/N–H stretching region between 3500–2900 cm^−1^. (b) LO–TO splitting of the ν_3_ vibrational mode of CO_2_. (c) Several new features formed including OCN^−^, CO & HCN and broad absorptions between 1750–1250 cm^−1^.

After deposition at 20 K, the CO_2_ : NH_3_ mixtures were irradiated with 1 keV electrons at discrete intervals cumulating in a total fluence of 3.37 × 10^16^ e^−^ cm^−2^. Fig. 1 shows the mid-IR spectra of a CO_2_ : NH_3_ mixture in a 1 : 1 ratio after set intervals of 1 keV electron irradiation. Mid-IR spectra of the irradiated ices in other ratios can be found in Section S2.2, Fig. S4 to S7 of the ESI.† For reference, the 1 keV electron irradiation mid-IR spectra of pure CO_2_ and pure NH_3_ are shown in Fig. S2 and S3 of the ESI,† respectively.

Irradiation with 1 keV electrons induced several changes within the 1 : 1 CO_2_ : NH_3_ mixture as shown in Fig. 1. The intensity of all the CO_2_ absorption bands decreased throughout electron irradiation. Within the O–H/N–H stretching region, between 3550–2900 cm^−1^ (Fig. 1a), an overall increase in intensity was observed with the appearance of shoulders on the higher wavenumber side of the NH_3_ ν_3_ absorption band and on the lower wavenumber side of the NH_3_ ν_1_ absorption band. A broad feature around 2800 cm^−1^ also appeared. The appearance of several distinct features was also observed in Fig. 1c: OCN^−^ (2170 cm^−1^), CO (2140 cm^−1^) and HCN (2092 cm^−1^). Several broad features also appeared between 1750–1250 cm^−1^: broad shoulders on both sides of the NH_3_ ν_2_ absorption band, and broad features centred around 1485 cm^−1^ and 1343 cm^−1^.

Differences between the stoichiometric ratios were observed, and the positions at which new features formed are listed in Table 2. Notably, the new absorption features due to electron irradiation in the region between 1800–1250 cm^−1^ were not present in all ratios and are discussed further in Section 3.2.1. The new absorption features which were present also varied in position. The ratio-dependent formation of CO and OCN^−^ are discussed in Section 3.2.2.

**Table 2:** Band assignments and positions of new features after 1 keV electron processing to a total fluence of 3.37 × 10^16^ e^−^ cm^−2^ of pure CO_2_ ice (1 : 0), pure NH_3_ ice (0 : 1) and CO_2_:NH_3_ mixtures (4 : 1, 2 : 1, 1 : 1, 1 : 2, 1 : 5 & 1 : 10) deposited at 20 K

Molecule	Vib. mode	Assignment	Ref.	Position (cm^−1^)							
				1 : 0	4 : 1	2 : 1	1 : 1	1 : 2	1 : 5	1 : 10	0 : 1
OCN^−^	ν_3_	C–N stretch	16			2170	2169	2166	2162	2159	
CO	ν_1_	C=O	17	2140	2140	2140	2140	2140	2140	2139	
HCN	ν_3_	C–N stretch	18					2092		2092	2095
CO^−^_3_	ν_1_	C=O stretch	19	2050							
		C=O stretch			1715	1720^*sh*^	1724^*sh*^	1718^*sh*^	1704^*sh*^		
		C=O stretch				1682^*sh*^	1689^*sh*^	1690^*sh*^			
								1583	1584	1583	
		COO^−^*asym*. stretch						1550	1551	1551	
								1500	1505	1508	
					1477	1481	1485				
		COO^−^*asym*. stretch						1380	1384	1381	
						1343	1340	1337	1339	1342	
		C–O stretch			1305	1302	1302	1300	1301		
O_3_	ν_3_	O–O *asym*. stretch	20	1040							

#### 3.2.1 New absorption peaks between 1800–1250 cm^−1^

**Fig. 2:**
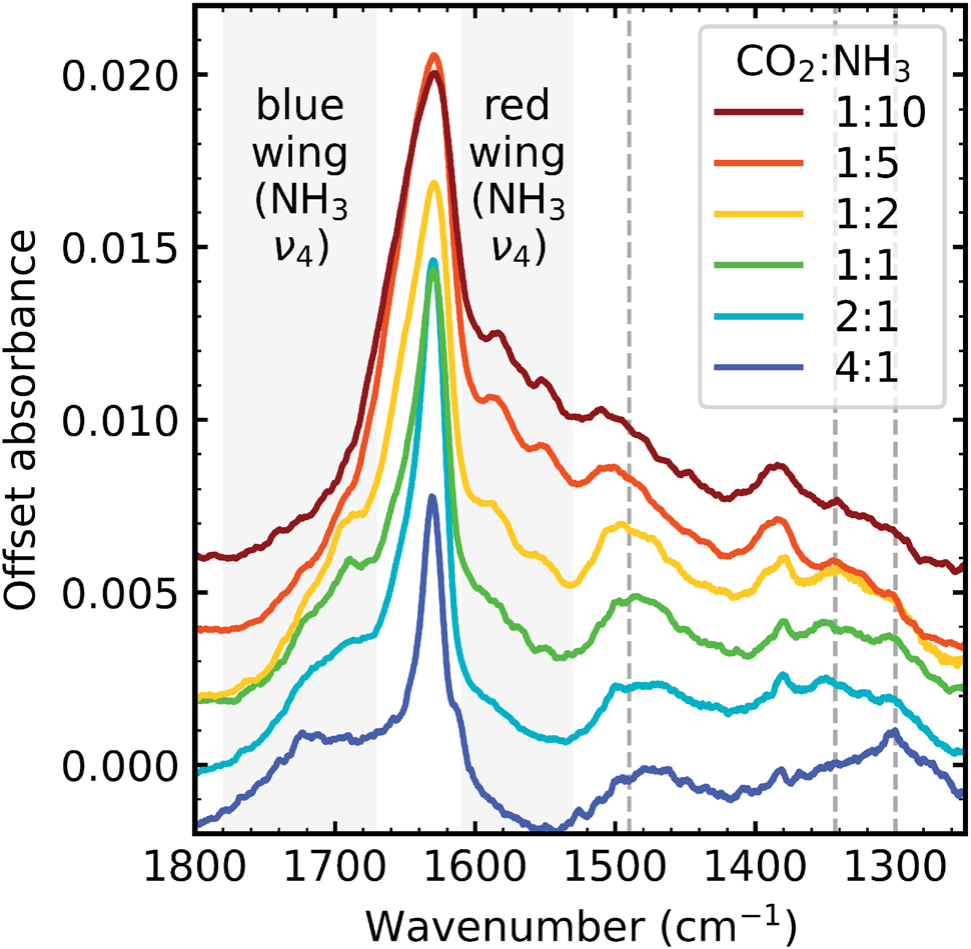
Mid-IR spectra of CO_2_ : NH_3_ mixtures (4 : 1, 2 : 1, 1 : 1, 1 : 2, 1 : 5 & 1 : 10) irradiated with 1 keV electrons at 20 K at a fluence of 3.37 ×10^16^ e^−^ cm^−2^. Grey dashed lines indicate newly formed, broad asymmetric absorption bands. Spectra are normalised to a thickness of 300 nm and offset on the *y*-axis for clarity.

Fig. 2 shows the region between 1800–1250 cm^−1^ for all ratios after irradiation with 1 keV electrons after a total fluence of 3.37 × 10^16^ e^−^ cm^−2^. The new absorption features formed are dependent on the stoichiometric ratios of the initial CO_2_ : NH_3_ mixtures. For example, on the blue wing of the NH_3_ ν_4_ absorption band one distinct absorption feature was identified in the 4 : 1 ratio at 1715 cm^−1^. For the 2 : 1, 1 : 1 & 1 : 2 ratios, the absorption feature on the blue wing of the NH_3_ ν_4_ absorption band was broad with two shoulder features. On the red wing of the NH_3_ ν_4_ absorption band, two distinct absorption features were observed near 1583 and 1550 cm^−1^ but only in the NH_3_-rich mixtures. Broad, asymmetric features were observed in the NH_3_-rich mixtures at ∼1505 cm^−1^ shifting to ∼1480 cm^−1^ in the other mixtures. Absorption features at ∼1380 cm^−1^ were observed for the NH_3_-rich mixtures. Broad absorption bands were observed for all ratios, except for the 4 : 1 ratio, near 1340 cm^−1^. An absorption feature was observed near ∼1300 cm^−1^ for all ratios except the 1 : 10 ratio.

Absolute band assignments of these irradiation features are very difficult without theoretical calculations and are beyond the focus of this paper. Broadly, the overall profile of the mixtures shown in Fig. 2 is similar for all ratios, except for the 4 : 1 ratio, indicative of products with similar functional groups. However, we do note that NH_3_-rich mixtures had additional absorption features at ∼1583 cm^−1^, ∼1550 cm^−1^, and ∼1380 cm^−1^ which could either indicate a more complex product, additional products or both.

#### 3.2.2 Formation of CO and OCN^−^

**Fig. 3:**
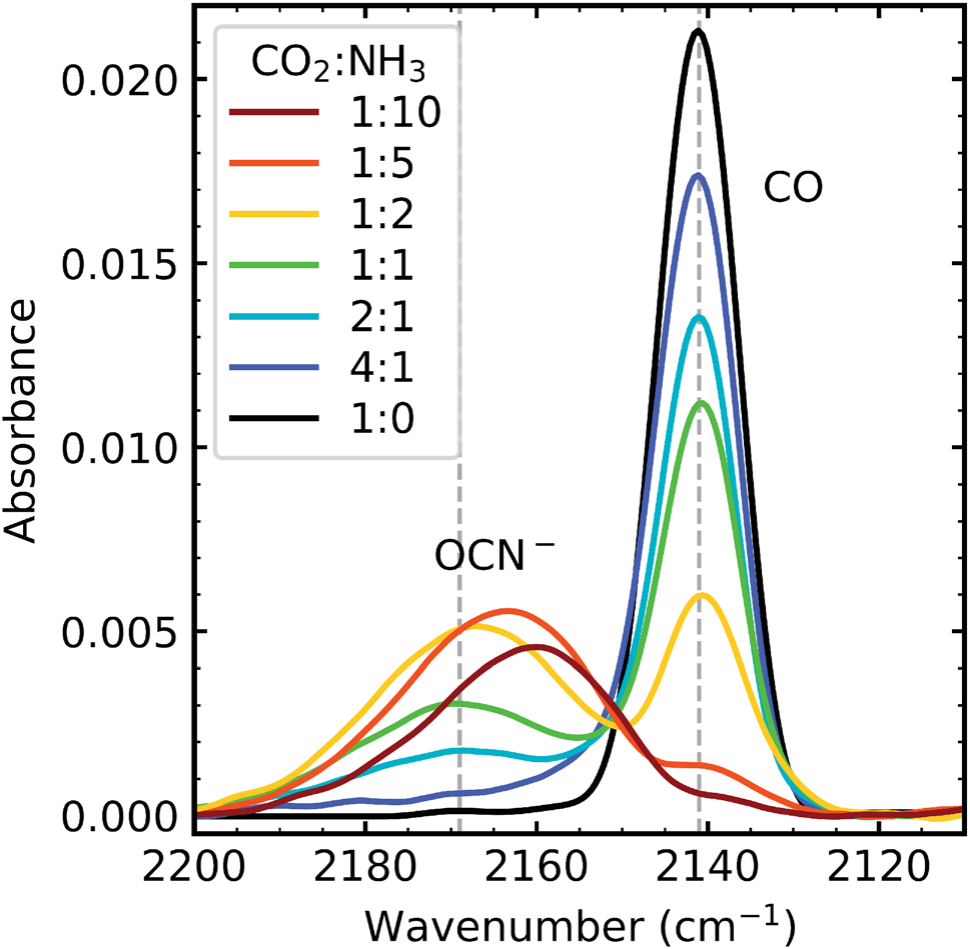
Mid-IR spectra of OCN^−^ and CO formed after irradiating CO_2_ : NH_3_ mixtures (4 : 1, 2 : 1, 1 : 1, 1 : 2, 1 : 5 & 1 : 10) with 1 keV electrons to a total fluence of 3.37 × 10^16^ e^−^ cm^−2^. Dashed lines indicate the positions of CO and OCN^−^ in the 1 : 1 ratio. Spectra are normalised to a thickness of 400 nm.

The formation CO and OCN^−^ was dependent on the initial mixing ratio as shown in Fig. 3, as was the relative amounts of CO and OCN^−^. CO forms from the direct dissociation of CO_2_ due to electron irradiation, and the amount of CO in the CO_2_ : NH_3_ ratios is in proportion to the concentration of CO_2_ within the mixtures. The 4 : 1 ratio had the highest amount of CO, and the 1 : 10 ratio had the lowest amount of CO. The formation of OCN^−^ occurs *via* several steps, first requiring CO formation.^4,5^ The highest amount of OCN^−^ formed was in the 1 : 5 ratio followed by, in descending order, the 1 : 2, 1 : 10, 1 : 1 & 2 : 1 ratios. While the amount of OCN^−^ seems to depend on the concentration of NH_3_, the 1 : 10 does not fit this trend. This suggests that the formation of OCN^−^ depends on an N-bearing intermediate but requires a minimum amount of CO, of which there is not enough in the 1 : 10 mixture. No detectable amounts of OCN^−^ were observed in the 4 : 1 ratio. The position of the OCN^−^ peak is also ratio-dependent, blueshifting as the concentration of NH_3_ decreases in the mixing ratios.

### 3.3 Thermal processing

**Fig. 4:**
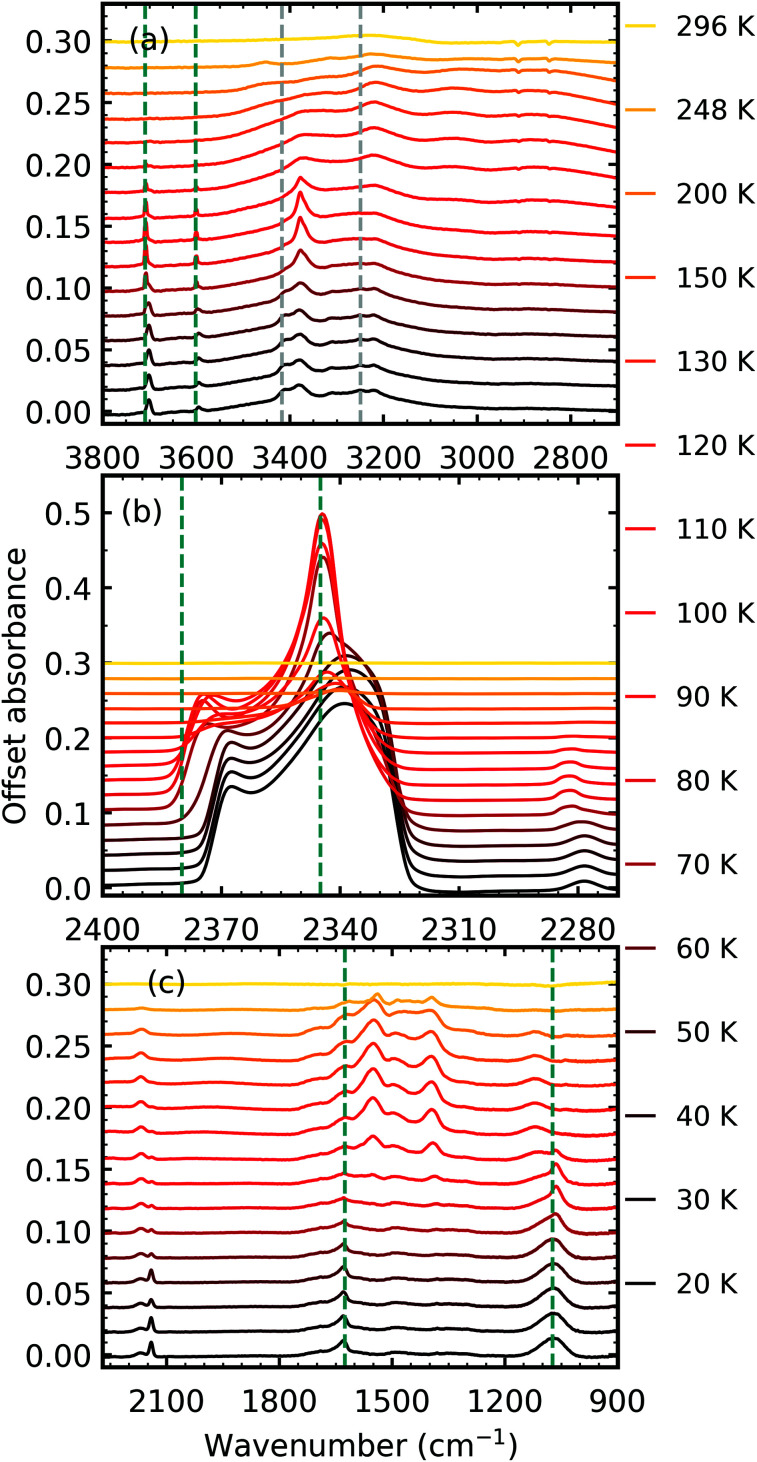
Example mid-IR spectra of thermal processing results of a CO_2_ : NH_3_ mixture in a 1 : 1 ratio after deposition and irradiation with 1 keV electrons to a fluence of 3.37 × 10^16^ e^−^ cm^−2^ at 20 K. Spectra are offset on the *y*-axis for clarity. See Section S2.3 in the ESI† for the mid-IR spectra of the 4 : 1, 2 : 1, 1 : 2, 1 : 5 & 1 : 10 ratios. (a) Segregation of the mixture was observed through the shift in position of the CO_2_ vibrational modes towards pure CO_2_ position which are indicated by blue dashed lines. Grey dashed lines indicate CO_2_ : NH_3_ molecular complex vibrational modes which disappeared between 60–70 K. (b) LO–TO splitting of the ν_3_ vibrational mode of CO_2_. Segregation of the mixture was observed through the shift in the position of the LO and TO modes towards pure CO_2_ positions which are indicated by blue dashed lines. (c) Thermally induced reaction at 80 K.

After 1 keV electron irradiation at 20 K, the CO_2_ : NH_3_ mixtures were thermally processed and analysed at discrete temperatures until desorption. Fig. 4 shows the mid-IR spectra of the thermal processing results of a CO_2_ : NH_3_ mixture in a 1 : 1 ratio. Mid-IR spectra of the other ratios can be found in Section S2.3 of the ESI.† Thermal processing after electron irradiation for pure CO_2_ and pure NH_3_ ices are given for reference in Fig. S8 and S9 of the ESI,† respectively.

Thermal processing, after electron irradiation, induced several changes within the 1 : 1 CO_2_ : NH_3_ mixtures as shown in Fig. 4. Similar to the non-irradiated 1 : 1 CO_2_ : NH_3_ mixture in RJ20, segregation of the homogeneous mixtures was identified through blue shifts in the CO_2_ vibrational modes towards pure CO_2_ wavenumbers between 60–70 K (Fig. 4a and b). Again similar to the non-thermally processed mixtures in RJ20, splitting of the ν_2_ fundamental mode of NH_3_ between 60–70 K signified a phase change in NH_3_. The CO peak area decreased throughout thermal processing until the CO desorbed between 130–150 K, whereas the OCN^−^ peak area increased until it reached a maximum near 130 K before decreasing until the residue material desorbed near 296 K. Between 70–80 K, a thermally induced reaction was initiated similar to the non-irradiated results in RJ20 with the absorption bands of the CO_2_ : NH_3_ molecular complex also disappearing at this temperature. While CO_2_ desorbed at a similar temperature in both the e-irradiated and non-irradiated 1 : 1 mixture of RJ20, the NH_3_ in the e-irradiated mixture desorbed at a higher temperature compared to NH_3_ in the non-irradiated mixture. A residue material was present after both CO_2_ and NH_3_ had desorbed with slight changes observed in the residue mixture between 150 and 200 K. The residue material from the e-irradiated mixtures desorbed at higher temperatures compared to the non-irradiated mixtures in RJ20.

The temperatures at which these changes occurred were dependent on the stoichiometric mixing ratio and are listed in Table 3.

**Table 3:** Summary of key observations during the thermal processing of e-irradiated CO_2_ : NH_3_ mixtures from mid-IR spectra

Observation	Ratio					
	4 : 1	2 : 1	1 : 1	1 : 2	1 : 5	1 : 10
CO_2_ segregation (K)	30–40	50–60	60–70	60–70	70–80	70–80
NH_3_ phase change (K)	30–40	60–70	60–70	70–80	70–80	70–80
Thermal reaction (K)	>90	>90	>90	>90	>90	>90
Disappearance of CO_2_ : NH_3_ complex (K)	30–40	50–60	60–70			
CO_2_ desorption (K)	100–110	131–150	120–130	120–130	120–130	120–130
NH_3_ desorption (K)	90–100	100–110	100–110	130–150	130–150	130–150
CO desorption (K)	80–90	110–121	130–150	130–150	110–120	70–80
OCN^−^ desorption (K)		223–250	248–296	250–298	250–268	250–285
Change in residue (K)	150–170	150–200	150–200	150–200	150–200	150–200
Residue desorption (K)	198–247	250–300	248–296	250–298	250–268	250–285

An overall comparison of the ratios from the e-irradiated CO_2_ : NH_3_ mixtures with the non-irradiated mixtures of RJ20 shows that the temperatures at which segregation of the CO_2_ : NH_3_ mixtures commenced and the onset phase change temperature of NH_3_ were very similar. The estimated electron penetration depth was at most 17%. Therefore, the observed shift in the CO_2_ absorption bands associated with segregation and the splitting pattern of the ν_3_ absorption band of NH_3_ associated with the onset of phase change of NH_3_ was likely representative of the non-irradiated part of the CO_2_ : NH_3_ ice samples.

The desorption temperatures of CO_2_ and NH_3_ were generally higher when subjected to electron irradiation compared to the desorption temperatures of CO_2_ and NH_3_ from the non-irradiated study of RJ20. Refractory material overlaying a more volatile material can elevate desorption temperatures due to trapping of molecules beneath the refractory layer^21,22^ and products formed from e-irradiation likely formed a more refractory layer in our ice mixtures.

### 3.4 Residue

In addition to e-irradiation induced reaction at 20 K from 1 keV electrons, a thermally induced reaction was observed for all CO_2_ : NH_3_ mixtures near ∼90 K. Electron irradiation introduces the formation of new molecules (*e.g.* CO and OCN^−^), but the electrons are estimated to only penetrate to a depth of 17% of the thickness of the ice. Although sputtering will reduce the ice thickness, the majority of the mixtures were still mainly composed of CO_2_ and NH_3_, as evidenced through the strong absorption bands of CO_2_ and NH_3_ observed after e-irradiation shown in Fig. 1 and S10–S14 of the ESI.† The thermal reaction present in the e-irradiated CO_2_ : NH_3_ mixtures was, therefore, likely to be the same as the non-irradiated ices in RJ20. Similar to RJ20, ammonium carbamate was identified *via* the COO^−^ asymmetric and symmetric stretches, and carbamic acid *via* the C–O and C=O stretches.

**Fig. 5:**
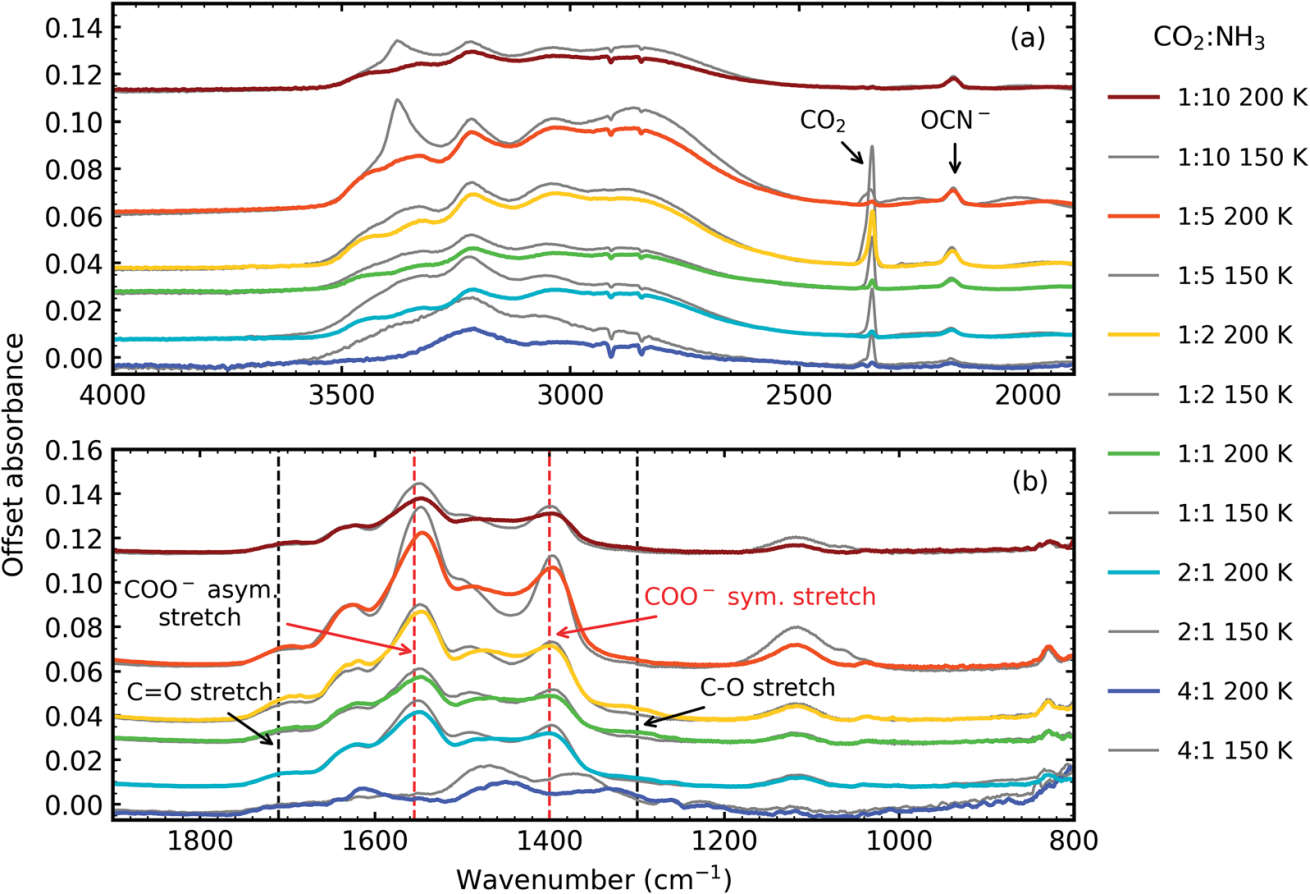
Mid-IR spectra of CO_2_ : NH_3_ ices thermally processed to 150 K (grey traces) and 200 K (coloured traces) after irradiation with 1 keV electrons at 20 K. (a) 4000–2500 cm^−1^ including the N–H and O–H stretching region (4000–2500 cm^−1^) and CO_2_ and OCN^−^ features (b) 1900–800 cm^−1^ with dashed lines indicating the COO^−^ asymmetric and symmetric stretches and C=O & C–O stretches. All spectra are normalised to a thickness of 400 nm and offset on the *y*-axis for clarity.

Fig. 5 shows the residue spectra at 150 K and 200 K for all CO_2_ : NH_3_ ratios. Ammonium carbamate and carbamic acid were identified in the residue spectra at 150 K and 200 K for all mixtures except the 4 : 1 ratio. Among these ratios, the 1 : 5 ratio had the highest amount of residue material, followed by the 1 : 2 and 1 : 1 ratios, with the 1 : 10 and 2 : 1 ratios forming the least amount of residue. Ammonium carbamate to carbamic acid conversion was observed between 150–200 K in all mixtures except the 4 : 1 ratio.

From Fig. 5 it is clear that the 4 : 1 residue at 150 K lacks the strong COO^−^ asymmetric and symmetric stretches suggesting that little to no ammonium carbamate formed, the overall residue spectral profile is different from the other ratios. Focussing on the region between 1900–800 cm^−1^, at 150 K, the 4 : 1 residue spectra has broad absorption peaks centred around 1470 cm^−1^ and 1368 cm^−1^ and a much weaker absorption peak at 1262 cm^−1^. Thermal processing to 200 K redshifts the broad peaks observed at 150 K to 1448 cm^−1^ and 1332 cm^−1^ and the weaker absorption peak to 1257 cm^−1^. The appearance of the absorption peak at 1612 cm^−1^ suggests thermal conversion occurred between 150 K and 200 K. While it is clear that the 4 : 1 residue was different from the other ratios, identification of this residue is not possible without further experimental and computational investigations.

**Fig. 6:**
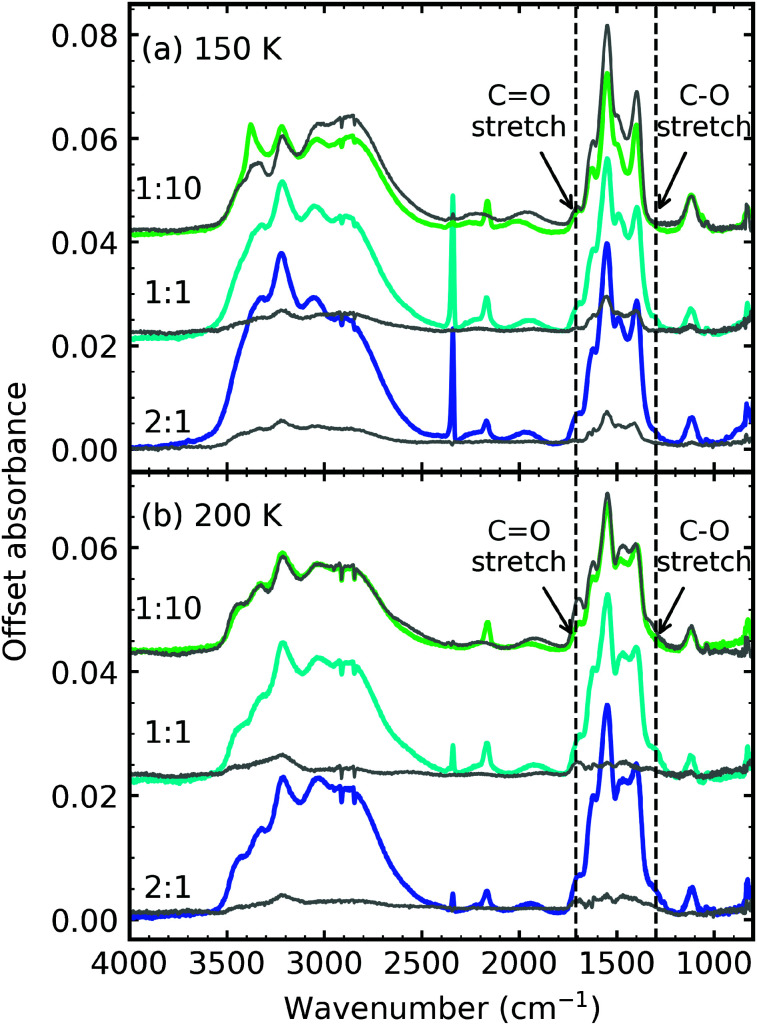
Comparison of the mid-IR residue spectra of the non-irradiated ices from RJ20 (grey traces) and e-irradiated ices of this paper (coloured traces) at (a) 150 K and (b) 200 K.

Fig. 6 shows the comparison between the residue spectra of the mid-IR 2 : 1, 1 : 1 & 1 : 10 ratios at 150 K and 200 K for the non-irradiated mixtures of RJ20 and the e-irradiated mixtures of this paper. The 1 : 10 residues at 150 K were remarkably similar in profile, suggesting that the thermally induced reaction, initiated at ∼90 K, was not significantly impacted by electron irradiation at 20 K. At 200 K, we observed a thermal conversion of ammonium carbamate to carbamic acid in the RJ20 1 : 10 residue through the increase in the C=O and C–O stretches at ∼1707 cm^-1^ and ∼1327 cm^−1^, respectively. However, very little thermal conversion of ammonium carbamate to carbamic acid was observed in the e-irradiated 1 : 10 ratio. Additionally, OCN^−^ was observed at 150 K and 200 K for the e-irradiated 1 : 10 residue. The e-irradiated residues at 150 K and 200 K were much more intense compared to the non-irradiated residues of RJ20, indicating the formation of higher amounts of residue material.

Electron irradiation at 20 K clearly influences the thermal reactivity of the 2 : 1 & 1 : 1 ratios. In the non-irradiated study of RJ20 the 1 : 1 and CO_2_-rich ratios formed lower amounts of residue material compared to the NH_3_-rich ratios. This was attributed to the different bonding environment of CO_2_ within the different CO_2_ : NH_3_ mixtures. CO_2_ dimers, which formed mainly in the CO_2_-rich and 1 : 1 mixtures, desorbed at lower temperatures compared to isolated CO_2_ within an NH_3_ matrix and CO_2_ : NH_3_ complexes found largely in NH_3_-rich mixtures. As discussed in Section 3.3, e-irradiation of the CO_2_ : NH_3_ mixtures appeared to elevate the desorption temperatures of CO_2_ and NH_3_ increasing the probability for a thermal reaction to occur before desorption of CO_2_ and NH_3_. Also, the residence of CO may play a part, with CO desorption delayed to higher temperatures in the 1 : 1 & 1 : 2 and 2 : 1 & 1 : 5 mixtures.

## 4 VUV results

### 4.1 Electron irradiation and thermal processing of pure CO_2_ and NH_3_

**Fig. 7:**
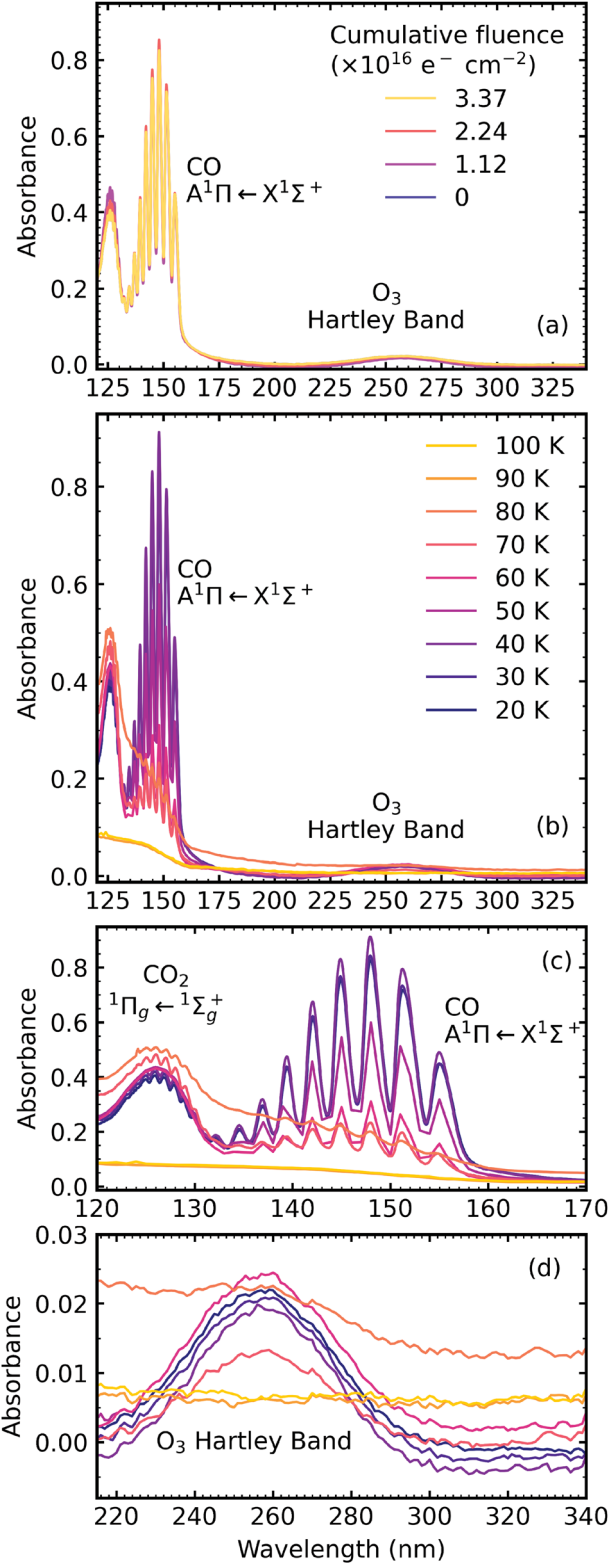
VUV spectra of pure CO_2_ deposited at 20 K (a) irradiated with 1 keV electrons at 20 K at discrete intervals to a total fluence of 3.37 × 10^16^ e^−^ cm^−2^; (b) thermally processed; (c) close-up of CO absorption band during thermal processing and (d) close-up of O_3_ absorption band during thermal processing.

**Fig. 8:**
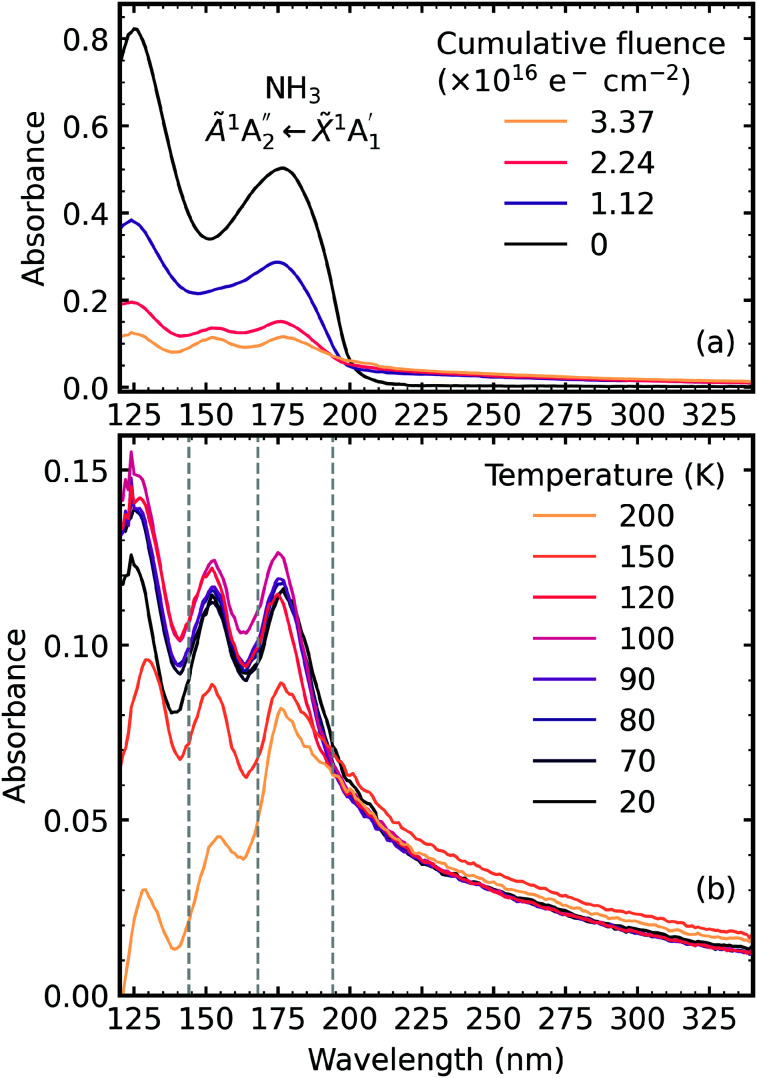
VUV spectra of pure NH_3_ deposited at 20 K (a) irradiated with 1 keV electrons at discrete intervals to a total fluence of 3.37 × 10^16^ e^−^ cm^−2^ and (b) thermally processed. Grey dashed lines indicate the absorption maxima for gas phase hydrazine.^34^

The VUV spectra of pure CO_2_ ice and pure NH_3_ ice deposited at 20 K and irradiated with 1 keV electrons at discrete intervals to a total fluence of 3.37 × 10^16^ e^−^ cm^−2^ are shown in Fig. 7a and 8a, respectively. We also present the subsequent thermal processing of the e-irradiated CO_2_ and NH_3_ ices in Fig. 7b–d and 8b, respectively.

Fig. 7a shows the result of e-irradiation of pure CO_2_ at 20 K with 1 keV electrons at discrete intervals. The formation of CO was observed by the appearance of an absorption band centred around 147 nm, which had intense vibrational structure, and was due to the A^1^Π ← X^1^Σ^+^ transition.^23–25^ We tentatively assign the observation of O_3_ to the broad absorption peak centred around 258 nm, which is known as the Hartley band.^26,27^ We note that this broad absorption peak may also be ascribed to the Cameron band of CO^28^ or a combination of both the Hartley band of O_3_ and the Cameron band of CO. However, interpretation of our e-irradiated CO_2_ : NH_3_ mixtures in Section 4.3, suggests that this band was more likely due to the presence of O_3_. After electron irradiation at 20 K pure CO_2_ was thermally processed, and VUV spectra were acquired at discrete temperatures until desorption. VUV spectra of the thermal processing of e-irradiated CO_2_ ice is shown in Fig. 7b with close-ups of the CO absorption band in Fig. 7c and O_3_ in Fig. 7d. The absorption band of CO due to the A^1^Π ← X^1^Σ^+^ transition disappeared between 80–90 K, as did the absorption band of the Hartley band of O_3_ with CO_2_ desorbing around 100 K.

Fig. 8a shows the irradiation of pure NH_3_ ice at 20 K with 1 keV electrons at discrete intervals. A new absorption feature centred around 150 nm was observed after an irradiation fluence of 1.12 × 10^16^ e^−^ cm^−2^. The intensity of the NH_3_ absorption peaks decreased significantly throughout electron irradiation. Due to the relatively strong VUV absorption cross-section of NH_3_ compared to CO_2_,^24^ a thinner ice sample was required to prevent saturation of the absorption peaks. The estimated electron penetration depth of the NH_3_ ice was 54% compared to the CO_2_ ice which was at 23%. After electron irradiation at 20 K, pure NH_3_ was thermally processed, and spectra were acquired at discrete temperatures until desorption. VUV spectra of the thermal processing of e-irradiated NH_3_ ice is shown in Fig. 8b. The e-irradiated absorption features shown in Fig. 8b persisted as a residue until 200 K. We believe a good candidate for this residue profile is hydrazine (N_2_H_4_). Previous studies identify hydrazine as a product of non-thermal processing (*e.g.* electrons, UV photons) of pure NH_3_ (ref. 29–32) with a desorption temperature between 155–172 K.^30,32,33^ The gas-phase VUV spectra of N_2_H_4_ and N_2_D_4_ are characterised by three absorption peaks with maxima near 144 nm, 168 nm & 194 nm.^34,35^ The three absorption peak maxima in Fig. 8b are at 129 nm, 152 nm & 176 nm, which may represent these the N_2_H_4_ bands since a shift in the absorption peaks from the gas phase to the solid phase is common.^24^

### 4.2 Deposition at 20 K

The following CO_2_ : NH_3_ mixtures were deposited at 20 K: 4 : 1, 2 : 1 & 1 : 3 and were the same as the ratios used in the VUV study of RJ20. For detailed characterisation of the CO_2_ : NH_3_ mixtures deposited at 20 K see Section 3.1 of RJ20. Deposition spectra are shown in Fig. S3.2 of the ESI.† Briefly, the absorption band due to the 
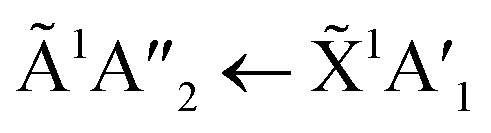
 electronic transition of NH_3_ largely overlaps and obscures the CO_2_ absorption bands due to the ^1^Π_g_ ← ^1^Σ_g_^+^ and ^1^Δ_u_ ← ^1^Σ_g_^+^ electronic transitions in the 1 : 3 ratio. However, in the 4 : 1 & 2 : 1 ratios the absorption band due to the ^1^Π_g_ ← ^1^Σ_g_^+^ electronic transition of CO_2_ is visible.

### 4.3 Electron irradiation at 20 K

**Fig. 9:**
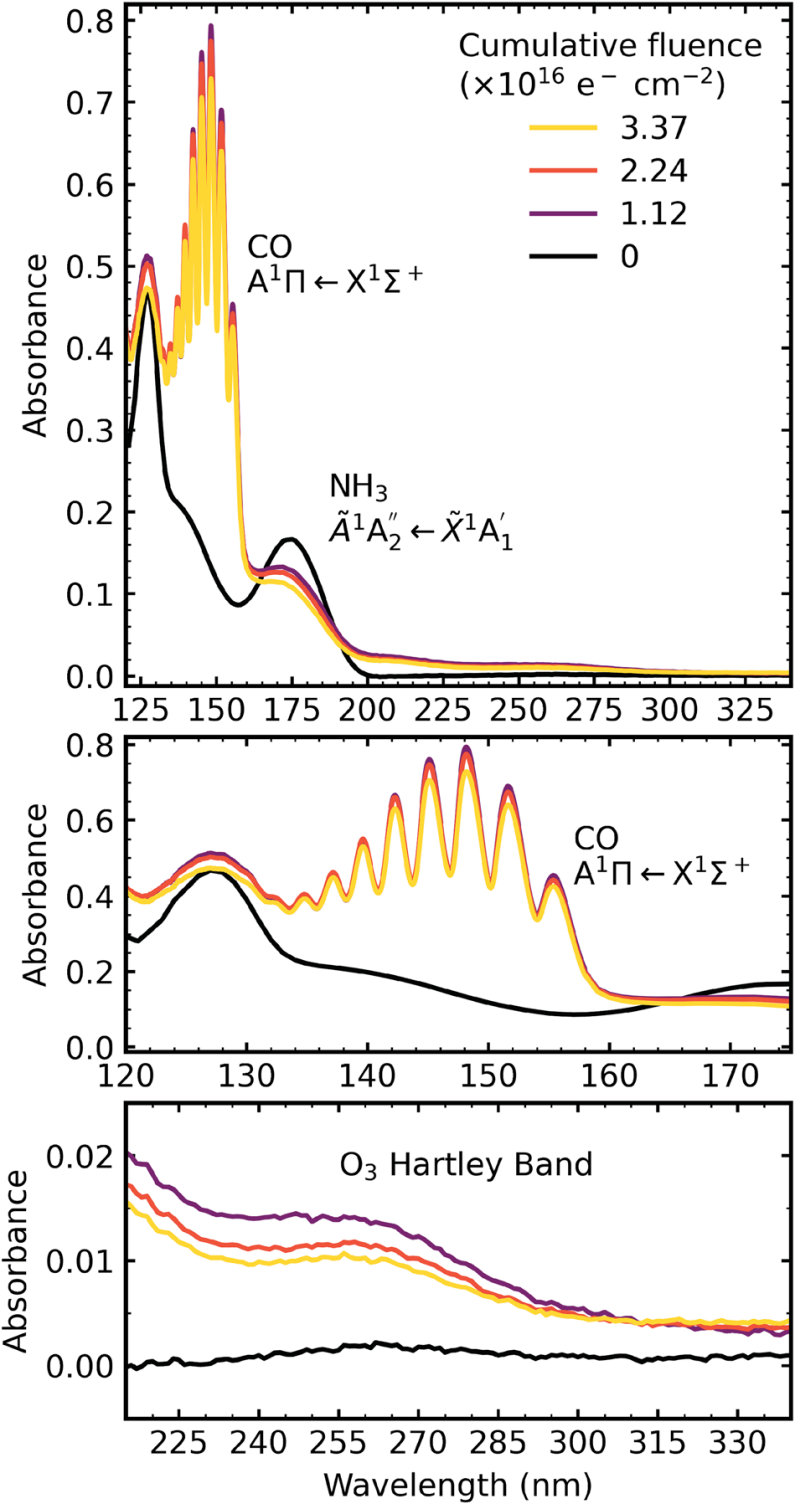
VUV spectra of a CO_2_ : NH_3_ mixture in a 4 : 1 ratio deposited at 20 K and then processed with electrons at discrete intervals to a total fluence of 3.37 × 10^16^ e^−^ cm^−2^. Middle panel shows a close-up of the region between 120–175 nm including the absorption band due to the CO A^1^Π ← X^1^Σ^+^ electronic transition. Bottom panel shows a close-up of the region between 220–340 nm including the O_3_ Hartley band.

**Fig. 10:**
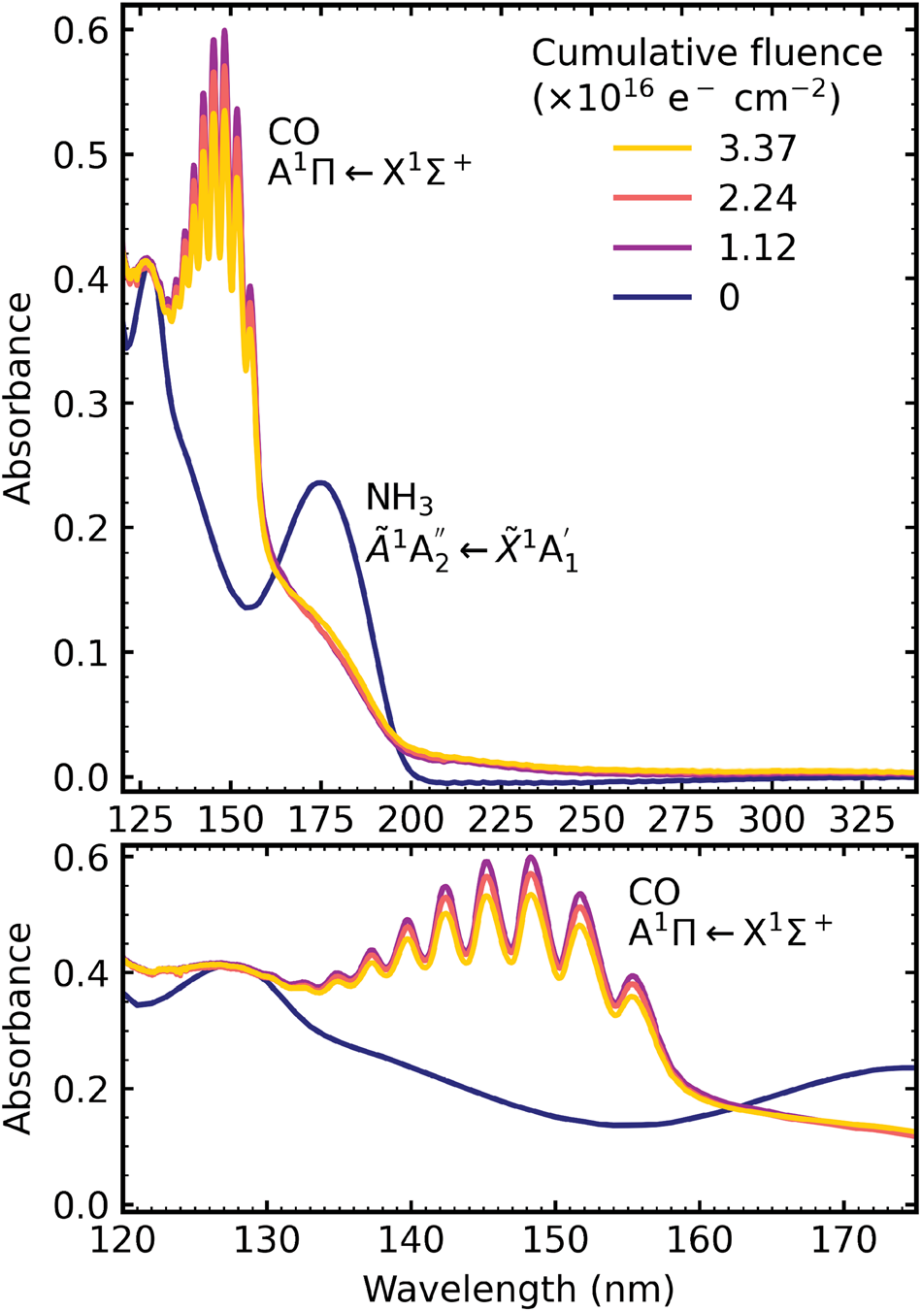
VUV spectra of a CO_2_ : NH_3_ mixture in a 2 : 1 ratio deposited at 20 K and then processed with electrons at discrete intervals to a total fluence of 3.37 × 10^16^ e^−^ cm^−2^. Bottom panel shows a close-up of the region between 120–175 nm including the absorption band due to the CO A^1^Π ← X^1^Σ^+^ transition.

**Fig. 11:**
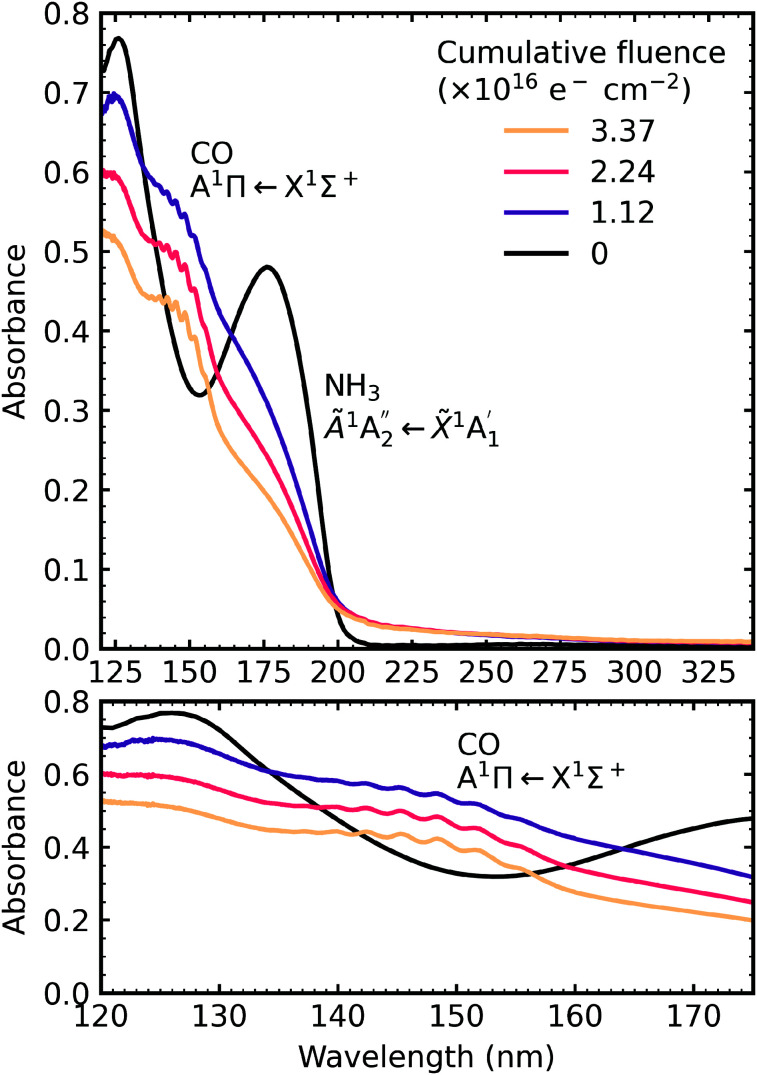
VUV spectra of a CO_2_ : NH_3_ mixture in a 1 : 4 ratio deposited at 20 K and then processed with electrons at discrete intervals to a total fluence of 3.37 × 10^16^ e^−^ cm^−2^. Bottom panel shows a close-up of the region between 120–175 nm including the absorption band due to CO A^1^Π ← X^1^Σ^+^ electronic transition.

After deposition at 20 K the CO_2_ : NH_3_ mixtures were irradiated with 1 keV electrons at discrete intervals. Fig. 9–11 show the VUV spectra of the electron irradiation of CO_2_ : NH_3_ mixtures in a 4 : 1, 2 : 1 & 1 : 3 ratio at 20 K, respectively.

Similar to the mid-IR spectra, the VUV spectra showed changes after electron irradiation. CO formed in all ratios, and was observed *via* the appearance of an absorption band centred around 147 nm, which was due to the A^1^Π ← X^1^Σ^+^ electronic transition. This transition had vibrational structure, which was most intense for the 4 : 1 ratio, followed by the 2 : 1 ratio and the 1 : 3 ratio. All of the ratios showed a decrease in the intensity of the absorption band due to the 
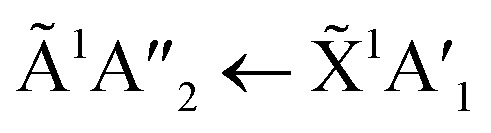
 electronic transition of NH_3_, with the excess NH_3_ ratio (1 : 3), having the highest relative decrease.

In addition, the 4 : 1 ratio had a broad absorption peak centred around 258 nm. We believe this absorption peak is due to the Hartley band of O_3_, rather than the Cameron band of CO. If the peak was due to the Cameron band of CO, we would expect to see it in the 2 : 1 ratio where relatively large amounts of CO were still observed. The formation of O_3_ was not observed in the mid-IR spectra due to the intense absorption peak of the ν_2_ absorption band of NH_3_ obscuring the O_3_ ν_3_ absorption peak at 1038 cm^−1^.

### 4.4 Thermal processing

**Fig. 12:**
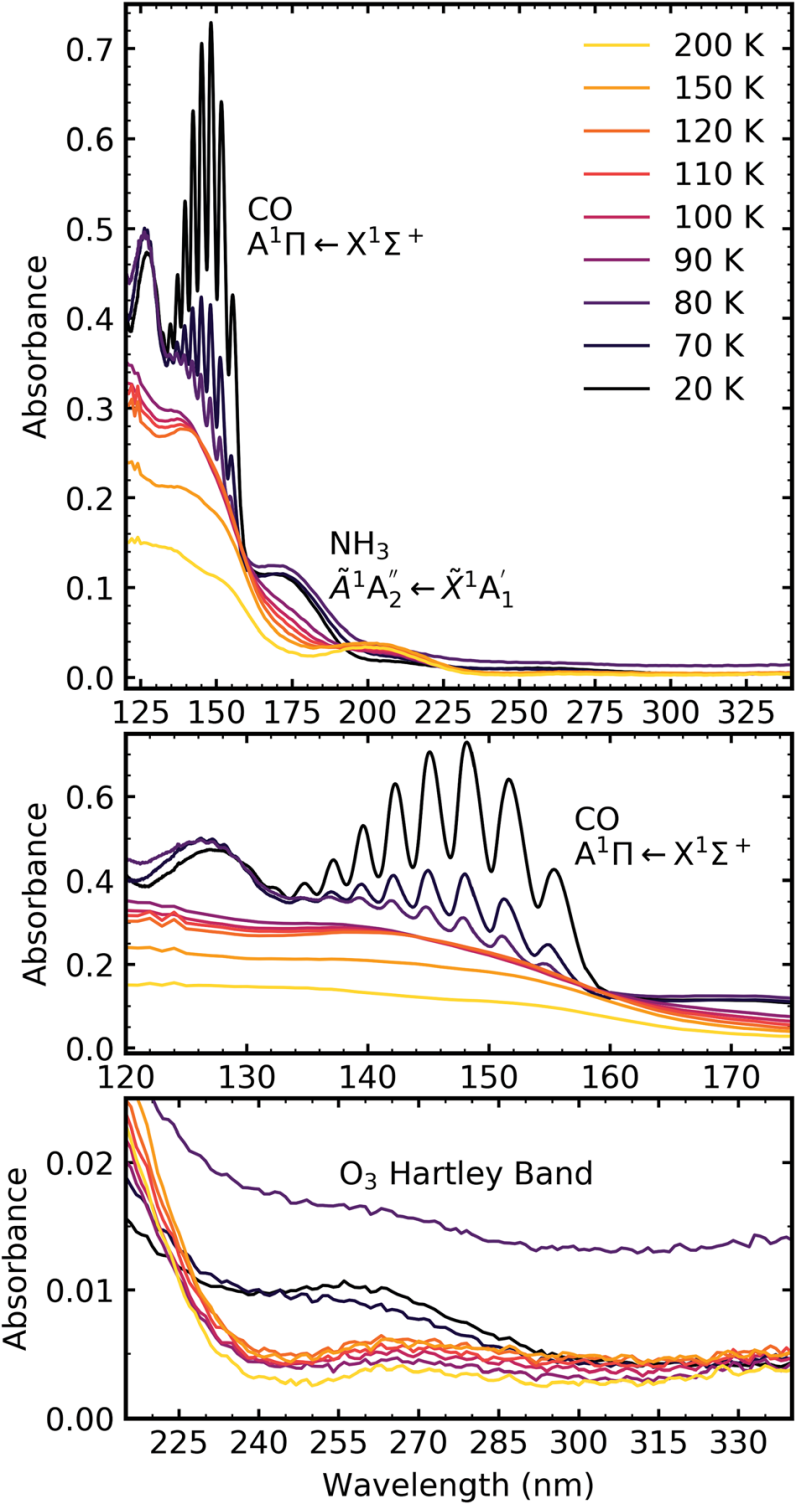
VUV spectra of the thermal processing of a 4 : 1 CO_2_ : NH_3_ mixture after 1 keV electron irradiation to a total fluence of 3.37 × 10^16^ e^−^ cm^−2^. Middle panel shows a close-up of the region between 120–175 nm including the absorption band due to the CO A^1^Π ← X^1^Σ^+^ electronic transition. Bottom panel shows a close-up of the region between 220–340 nm including the O_3_ Hartley band.

**Fig. 13:**
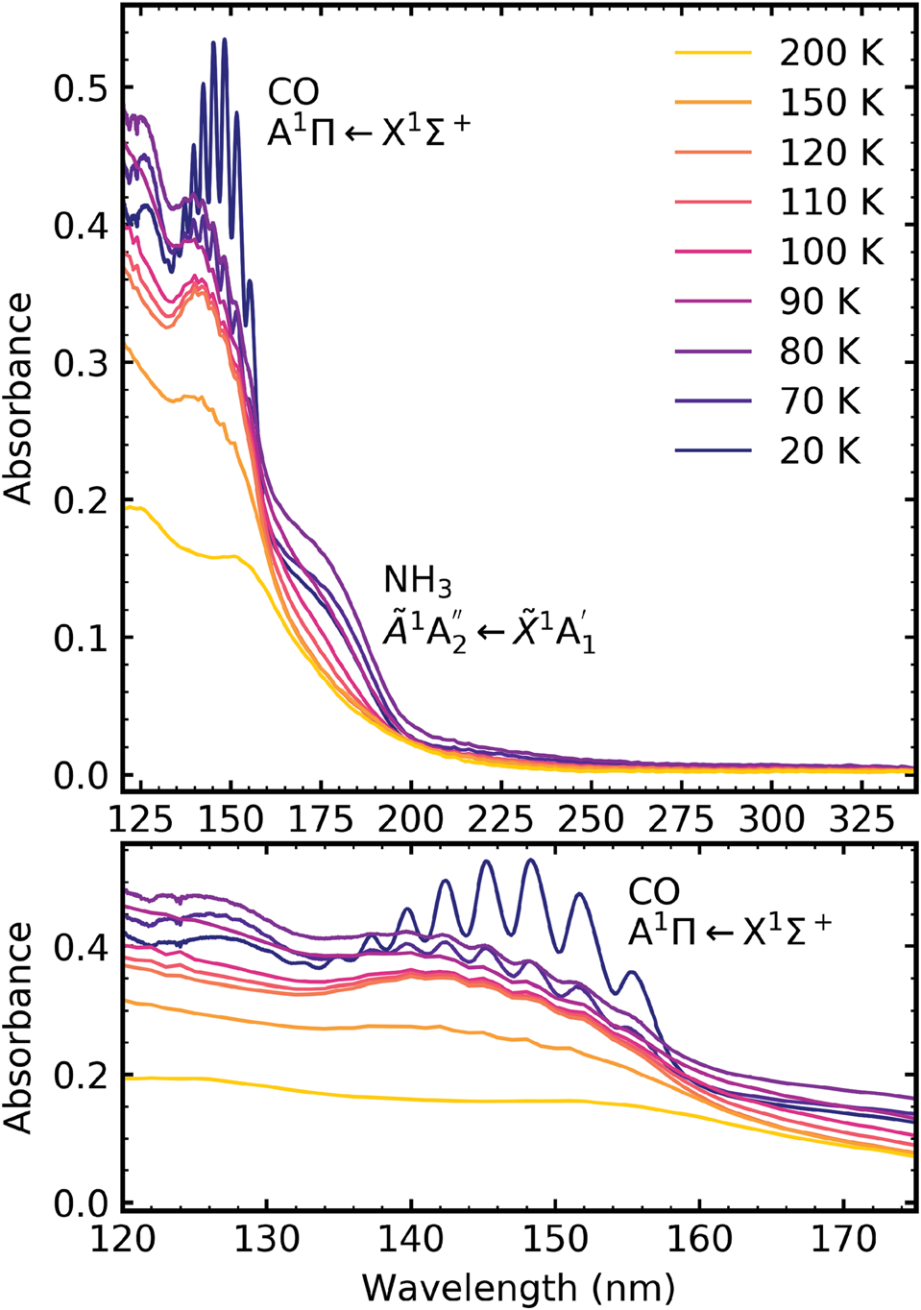
VUV spectra of the thermal processing of a 2 : 1 CO_2_ : NH_3_ mixture after 1 keV electron irradiation to a total fluence of 3.37 × 10^16^ e^−^ cm^−2^. Bottom panel shows a close-up of the region between 120–175 nm including the absorption band due to the CO A^1^Π ← X^1^Σ^+^ electronic transition.

**Fig. 14:**
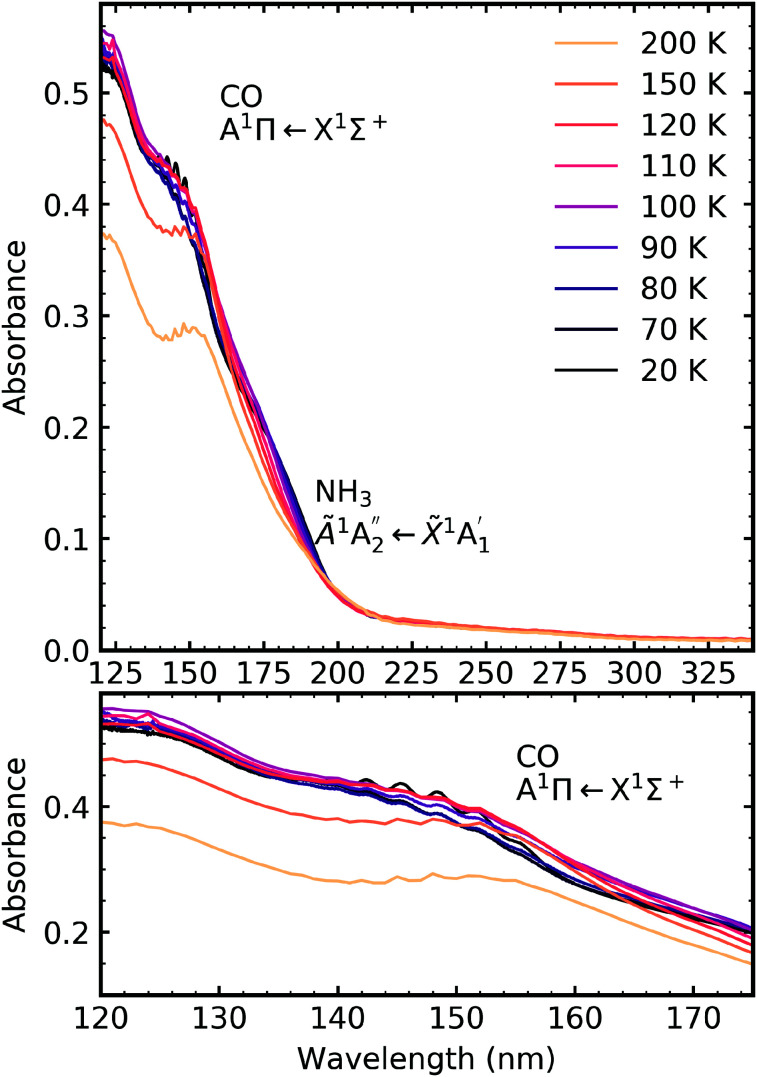
VUV spectra of the thermal processing of a 1 : 3 CO_2_ : NH_3_ mixture after 1 keV electron irradiation to a total fluence of 3.37 × 10^16^ e^−^ cm^−2^. Bottom panel shows a close-up of the region between 120–175 nm including the absorption band due to CO A^1^Π ← X^1^Σ^+^ electronic transition.

After electron irradiation at 20 K the CO_2_ : NH_3_ mixtures were thermally processed and VUV spectra were measured at discrete temperatures until desorption. Fig. 12–14 show the VUV thermal processing spectra of the e-irradiated 4 : 1, 2 : 1 & 1 : 3 ratios, respectively.

For the 4 : 1 ratio, the VUV spectra changed quite significantly between 80–90 K. Desorption of CO was marked by the disappearance of the intense vibrational structure on the absorption band due to the A^1^Π ← X^1^Σ^+^ electronic transition between 80–90 K corroborating the mid-IR results shown in Section 3.3 and consistent with the CO_2_ desorption temperature in pure irradiated CO_2_ ice. The Hartley band of O_3_ also disappeared at the same temperature as CO, indicating O_3_ desorption. The absorption band due to the 
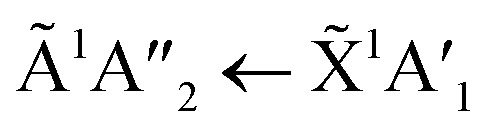
 electronic transition of NH_3_ also decreased significantly between 80–90 K and an absorption band centred around 205 nm also formed at this temperature, indicating a thermal reaction, which continued to increase in intensity until 150 K.

The thermal processing of the VUV spectra for the 2 : 1 and 1 : 3 ratios did not change as significantly as the 4 : 1 ratio. The vibrational structure on the absorption band of CO due to the A^1^Π ← X^1^Σ^+^ electronic transition disappeared between 110–120 K for the 2 : 1 ratio and 120–150 K for the 1 : 3 ratio, corroborating the mid-IR results presented in Section 3.3. The absorption band due to the 
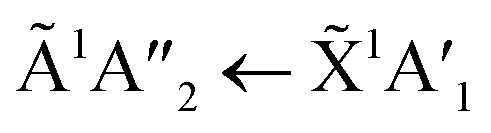
 electronic transition of NH_3_ gradually decreased throughout thermal processing until 150 K where it disappeared for the 2 : 1 ratio. For the 1 : 3 ratio, the absorption band due to the 
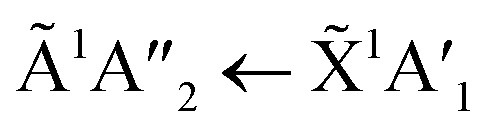
 electronic transition of NH_3_ had mostly disappeared after electron irradiation at 20 K before thermal processing. At 150 K, an absorption peak was observed centred around 145 nm in the 2 : 1 & 1 : 3 ratios had vibrational structure. At 200 K, the vibrational structure disappeared in the 2 : 1 ratio but remained in the 1 : 3 ratio. This vibrational structure was not associated with CO, which had desorbed between 110–150 K.

In RJ20, a phase change of NH_3_ was observed *via* the formation of a Wannier–Mott exciton peak at 194 nm in the 1 : 3 ratio when thermally processed to 90 K. However, no Wannier–Mott exciton was observed in the e-irradiated 1 : 3 ratio. The Wannier–Mott exciton peak intensity is associated with the presence of many NH_3_ crystallite grain boundaries (*i.e.*, smaller crystallites). Due to electron irradiation, about 42% of the 1 : 3 ratio ice sample was processed, which reduced the number of pure NH_3_ crystallite grain boundaries in the non-irradiated part of the sample, and hence there was no observation of the NH_3_ Wannier–Mott exciton peak.

### 4.5 Residue

**Fig. 15:**
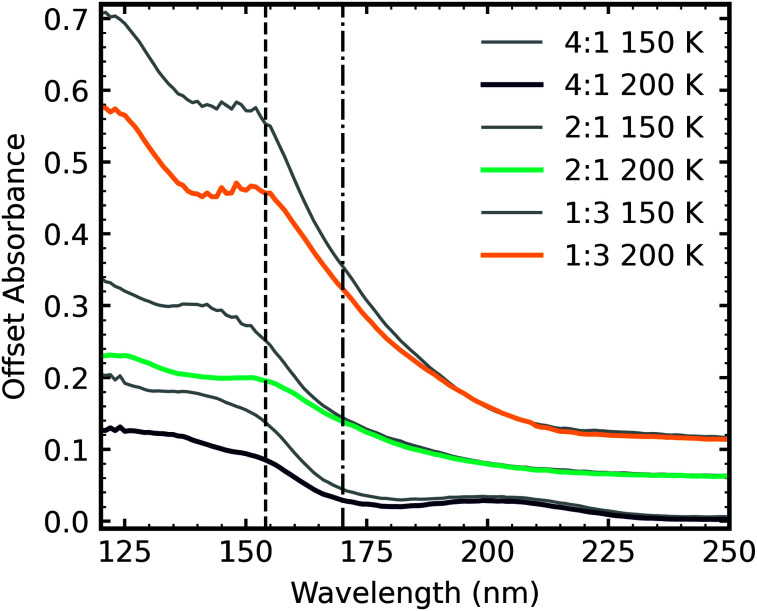
VUV residue spectra of CO_2_ : NH_3_ mixtures at 150 K and 200 K after 1 keV electron irradiation at 20 K and subsequent thermal processing. The dash dot line highlights a feature at 152 nm and the dashed line highlights a feature observed at 170 nm in RJ20. Spectra are normalised to a thickness of 200 nm and offset on the *y*-axis for clarity.

Ammonium carbamate and carbamic acid were identified at 150 K and 200 K for all mid-IR ratios except the 4 : 1 ratio, which exhibited a different residue spectrum (Fig. 5). Fig. 15 shows the VUV residue spectra at 150 K and 200 K for the 4 : 1, 2 : 1 & 1 : 3 ratios. At 150 K and 200 K, the 1 : 3 residue profiles were similar. Although, the intensity of the absorption peaks centred around 125 nm and 150 nm decreased at 200 K. A slight redshifting of the 150 nm peak to 152 nm also occurred. The 2 : 1 residue has an absorption peak centred around 145 nm at 150 K, which disappeared at 200 K, revealing a peak centred around 154 nm. A peak around 125 nm resolved upon thermal processing. The 4 : 1 residue has peaks centred around 145 nm and 205 nm at 150 K. The peak at 145 nm decreased in intensity upon thermal processing to 200 K, but the peak centred around 205 nm remained at a similar intensity.

**Fig. 16:**
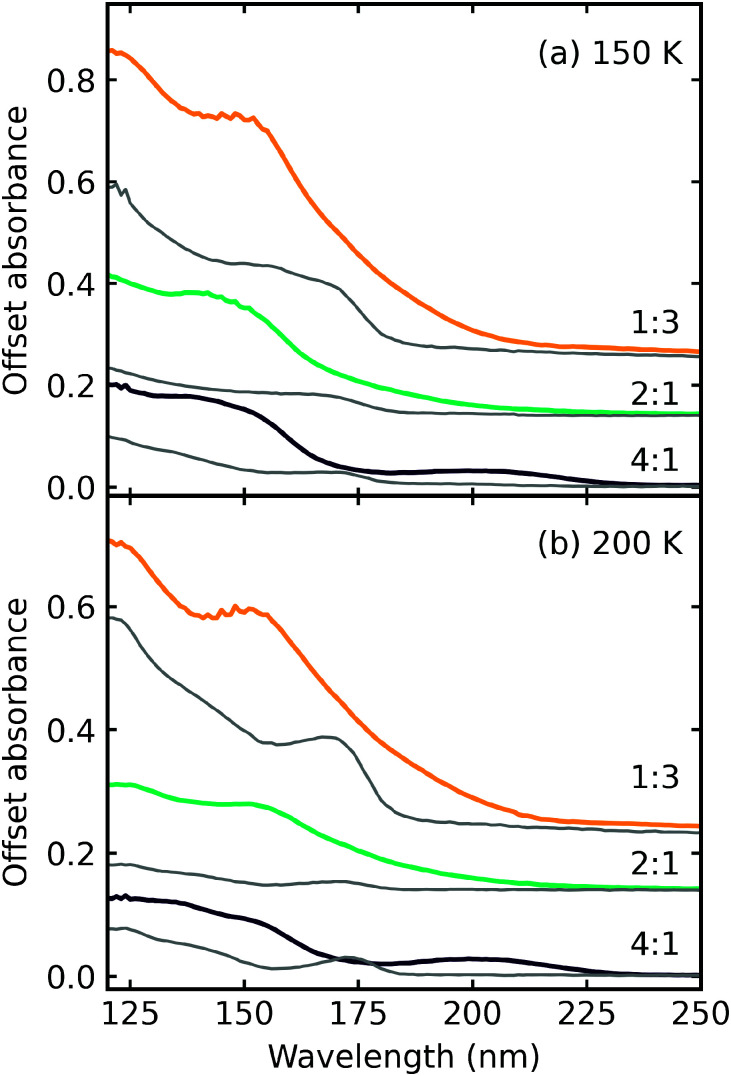
Comparison of the VUV residue spectra of the non-irradiated ices from RJ20 (grey traces) and e-irradiated ices of this paper (coloured traces) at (a) 150 K and (b) 200 K for different stoichiometric ratios of CO_2_ : NH_3_ mixtures.

Fig. 16 shows a direct comparison between the VUV 4 : 1, 2 : 1 & 1 : 3 residue spectra of the CO_2_ : NH_3_ residues at 150 K and 200 K of the non-irradiated study of RJ20 and the e-irradiated mixtures in this study. In agreement with the mid-IR residue spectra comparison (Fig. 6), the residue spectra for the e-irradiated residues were more intense than the non-irradiated VUV residue spectra of RJ20. At both 150 K and 200 K, the e-irradiated residues lacked the absorption peak at ∼175 nm observed in the RJ20 residues. Similar to the 4 : 1 mid-IR residue spectra, the 4 : 1 ratio of the e-irradiated residue has a different spectral profile to the equivalent RJ20 ratio.

A tentative assignment of the absorption peaks was given in RJ20 and summarised here. Tentatively, an absorption peak at ∼150 nm was assigned as arising due to an electronic transition of ammonium carbamate, and an absorption peak at ∼170 nm was assigned as arising due to an electronic transition of carbamic acid. No absorption peak was observed at ∼170 nm for the e-irradiated 2 : 1 and 1 : 3 residues, further supporting the assignment of carbamic acid being responsible for the transition at ∼175 nm. Small amounts of carbamic acid were probably present within the 2 : 1 and 1 : 3 residues but obscured by the ammonium carbamate transition at ∼150 nm. The absence of an absorption peak at ∼170 nm in the e-irradiated 4 : 1 ratio also confirmed the mid-IR results of Section 3.4 which showed no evidence of the formation of carbamic acid. The e-irradiated residue spectra at 200 K for all mixtures contained a transition at ∼150 nm. This transition was tentatively assigned as being due to ammonium carbamate in RJ20. No strong COO^−^ stretches were present in the mid-IR 4 : 1 residues (Fig. 5), suggesting that ammonium carbamate was not present in this mixture.

### 4.6 Rayleigh scattering tails

Rayleigh scattering tails are observed in VUV spectra when particle sizes are less than *λ*\10, and the intensity of scattered light is proportional to *λ*^−4^. Similar to RJ20, Rayleigh scattering tails were observed because of the rough, clumpy surface of the ice film as opposed to scattering tails observed in astrophysical ices, which did not fully wet the surface.^36,37^

The Rayleigh scattering tails were fitted using the following model introduced in RJ20:1
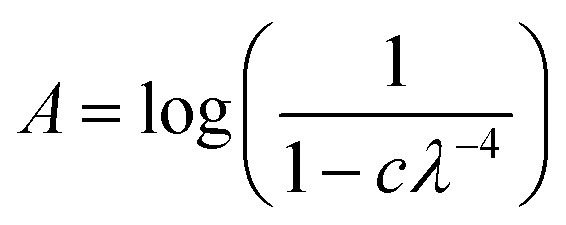
where *c* is a constant of proportionality and is dependent on the particle size, the refractive index and also the number density of scatterers present within the sample.

Similar to RJ20 we present the Rayleigh scattering tails as a fractional change in the constant of proportionality of the processed ice relative to the constant of proportionality at deposition (Δ*c*):2
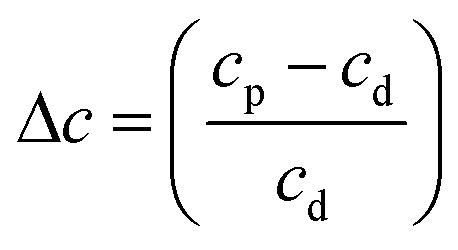
where *c*_p_ is the constant of proportionality of the processed (electron or thermal) ice sample and *c*_d_ is the constant of proportionality of the ice sample at 20 K.

**Fig. 17:**
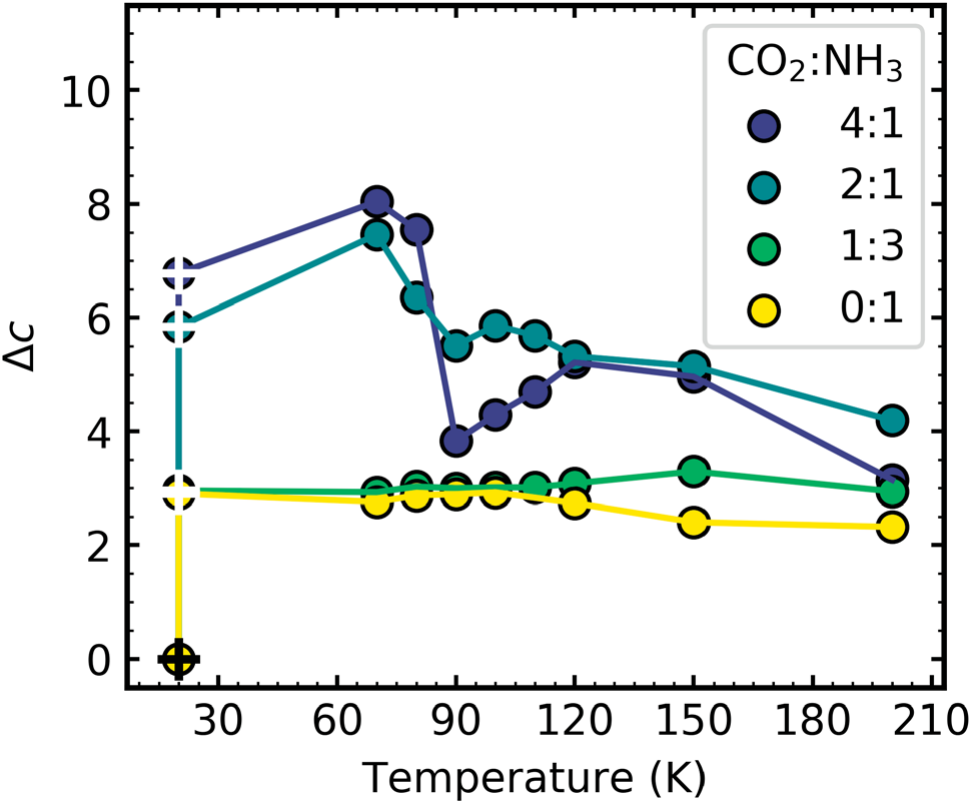
Comparison of the fractional change (Δ*c*) in the constant of proportionality of the processed ice (*c*_p_) relative to the constant of proportionality at deposition (*c*_d_) as a function of temperature after electron irradiation with 1 keV electrons. Two scatter points are shown at 20 K for each ratio, the scatter points marked with the black cross hairs indicate the Δ*c* at 20 K before electron irradiation, the scatter point with the white cross hair indicates the Δ*c* after 1 keV e-irradiation to 3.37 × 10^16^ e^−^ cm^−2^. The Δ*c* values are normalised to a thickness of 200 nm.

Fig. 17 shows the thermal evolution of Δ*c* after 1 keV electron irradiation for pure NH_3_ (0 : 1) and the CO_2_ : NH_3_ mixtures (4 : 1, 2 : 1 & 1 : 3). The scattering tails for pure CO_2_ were outside the Rayleigh scattering regime. In Fig. 17 there are two scatter points marked at 20 K for each ratio. The white crosshair scatter points indicate the Δ*c* values after 1 keV electron irradiation to a total fluence of 3.37 × 10^16^ e^−^ cm^−2^. For all CO_2_ : NH_3_ ratios and pure NH_3_ the Δ*c* value was significantly higher after electron irradiation compared to before deposition. Molecular dissociation and the formation of new products due to electron irradiation of the samples appeared to disrupt the ice structure. These VUV scattering results provide direct evidence that as well as inducing chemical changes, electron irradiation also caused macroscopic changes in ice structure and morphology. A significant decrease in the Δ*c* value at 90 K, for the 4 : 1 ratio, and to a lesser extent the 2 : 1, corresponded to the combined desorption of CO and CO_2_, consequently leading to rearrangement within the ice. The 1 : 3 had the least amount of CO_2_ and CO and their desorption appeared to have little effect on the Δ*c* value. The Δ*c* value of pure NH_3_ also remained relatively constant throughout thermal processing after electron irradiation at 20 K.

## 5 Discussion

We set out with the aim of demonstrating the impact that the different chemical and physical properties of a range of stoichiometric CO_2_ : NH_3_ ice mixtures can have on the molecular synthesis induced by electron irradiation and subsequent thermal processing.

In agreement with three out of four previous studies on the non-thermal processing of CO_2_ : NH_3_ ices below 16 K, we observed the formation of CO and OCN^−^ after 1 keV electron irradiation at 20 K.^4–6^ Bertin *et al.* did not observe CO or OCN^−^ after 9–20 eV electron irradiation at 30 K and noted that if CO had formed, it would have desorbed at their working temperature of 30 K. Given that OCN^−^ requires the formation of CO, this would explain the lack of OCN^−^.^4,5^ For the other products reported in previous studies, *i.e.* ammonium carbamate, ammonium formate, carbamic acid, NH^+^_4_ and N_2_O, most of their identifying functional groups are within the 3500–2800 cm^−1^ or 1800–1250 cm^−1^ regions where significant overlap occurs with the strong NH_3_ absorption bands. We find it difficult to make absolute assignments of the IR absorption peaks within these two regions. Overlap from the intense NH_3_ absorption bands, similar functional groups present within molecules (*e.g.* ammonium carbamate and ammonium formate), and matrix-isolation effects due to the different stoichiometric ratios of CO_2_ : NH_3_ mixtures are all contributing factors. As such, we do not identify molecules within this region at e-irradiation temperatures.

Our e-irradiation of stoichiometric ratios revealed that the 4 : 1 ratio formed O_3_, not observed in the other ratios. O_3_ formation in pure CO_2_ is a multi-step process which is summarised below:

3 CO_2_ → CO + O

4 O + O → O_2_

5 O_2_ + O → O_3_

Within the CO_2_ : NH_3_ mixtures, the only source of O atoms comes from the CO_2_ and therefore it is not surprising that O_3_ was formed in CO_2_ : NH_3_ mixture with the highest amount of CO_2_. However, compared to the O_3_ absorption peak in the pure CO_2_ ice, the O_3_ absorption peak in the 4 : 1 ratio is much weaker, indicating a lower amount of O_3_ formed in the 4 : 1 ratio compared to the pure CO_2_ ice. Apart from there being fewer O atoms available due to less CO_2_, other reactions occurring due to the presence of NH_3_ and its e-irradiated products in the mixtures will reduce the amount O atoms available for O_3_ formation. It appears that for the other CO_2_ : NH_3_ mixtures, O-containing molecules like OCN^−^ are formed either preferentially over O_3_ during e-irradiation or deplete the available O-atoms before O_3_ can form.

Our VUV spectroscopic study was particularly useful for observing the formation of O_3_, which was otherwise obscured by the intense ν_2_ absorption band of NH_3_ in the mid-IR study. Other than the identification of CO, OCN^−^ and O_3_, no identification of products due to e-irradiation was made. Mid-IR spectra obtained for the stoichiometric mixing ratios of CO_2_ : NH_3_ indicated a ratio-dependence on products formed from e-irradiation with NH_3_-rich mixtures showing more complex spectra within the region between 1800–1200 cm^−1^.

Comparison of our 1 : 1 CO_2_ : NH_3_ residue results with the residue of Jheeta *et al.*, who also used 1 keV electrons to irradiate their 1 : 1 CO_2_ : NH_3_ mixture albeit at a slightly higher temperature of 30 K, showed good agreement.^4^ While Jheeta *et al.* only identified ammonium carbamate, an absorption peak at ∼1700 cm^−1^ is indicative of a C=O stretch suggesting that carbamic acid was also present. Our 1 : 1 results were also in agreement with the 1 : 1 residues of Bossa *et al.*^5^ and Bertin *et al.*^3^ who identified ammonium carbamate and carbamic acid within their residue material. No comparison was made with Lv *et al.*^6^ as they did not thermally process their irradiated CO_2_ : NH_3_ mixtures.

By combining our e-irradiated results with our non-irradiated thermal study of CO_2_ : NH_3_ mixtures, presented in RJ20, we were able to further elucidate the impact of electron irradiation. Excluding the 4 : 1 ratio, all of the other e-irradiated CO_2_ : NH_3_ ratios formed a similar residue to the non-irradiated residues in RJ20. This suggests that the e-irradiated residues, apart from the 4 : 1 residue, are comprised of products mainly induced by the thermal reaction occurring at approximately 80–90 K. However, higher amounts of residue material formed in the e-irradiated CO_2_ : NH_3_ mixtures compared to the non-irradiated mixtures of RJ20. We believe this was due to the refractory layer formed from electron irradiation at 20 K, which consequently elevated the desorption temperature of CO_2_, thus allowing more time for a thermal reaction to occur. This corresponds to the practices within the literature which use more refractory molecules as ‘plugs’ to prevent the desorption of more volatile molecules.^21,22^

We also observed that less conversion of ammonium carbamate to carbamic acid occurred in the e-irradiated CO_2_ : NH_3_ mixtures upon thermal processing between 150–200 K. For example, the non-irradiated CO_2_ : NH_3_ 2 : 1, 1 : 1, 1 : 3 & 1 : 10 mixtures showed an increase in the C=O and C–O stretch in Fig. 5 of RJ20, whereas, very little change was observed in Fig. 5 for the thermally processed e-irradiated CO_2_ : NH_3_ mixtures. Ammonium carbamate appears to be stabilised by the products in the e-irradiated mixtures. At 150 K and 200 K, the e-irradiated product of OCN^−^ is present, which we suggest stabilises the ammonium carbamate, thus preventing decomposition to carbamic acid at higher temperatures.

The Rayleigh scattering tails obtained from VUV spectra gave direct evidence of physical changes within the CO_2_ : NH_3_ ice mixtures due toelectron irradiation.

## 6 Conclusions

We systematically investigated the effect of the stoichiometric mixing ratio on 1 keV e-irradiated CO_2_ : NH_3_ ices and subsequent thermal processing using mid-IR and VUV spectroscopy. Our work is the first time that a study has focussed on the non-thermal processing of stoichiometric mixing ratios of CO_2_ : NH_3_ ices. We show that the small e-irradiation products of CO and OCN^−^ are strongly dependent on the initial mixing ratio of CO_2_ and NH_3_, with no observable OCN^−^ formed in the 4 : 1 ratio. However, the 4 : 1 ratio did form O_3_ after e-irradiation at 20 K which was not observed in the other CO_2_ : NH_3_ mixtures. We also show that the CO_2_-rich, e-irradiated 4 : 1 CO_2_ : NH_3_ ratio formed a markedly different residue upon thermal processing compared to the other ratios. The other ratios formed similar residues between themselves and similar residues to their thermal processing counterpart residues from RJ20. However, ammonium carbamate to carbamic acid conversion was arrested in the e-irradiated residues. The astrophysical implications of these results, along with the results of RJ20, will be discussed in a forthcoming paper.

## Conflicts of interest

There are no conflicts to declare.

## Supplementary Material

RA-011-D1RA05600J-s001
