# Supplementary material for: Systematic investigation of CO2 : NH3 ice mixtures using mid-IR and VUV spectroscopy – part 2: electron irradiation and thermal processing
Source: RSC Adv. 2021 Oct 7;11(52):33055–69. doi: 10.1039/d1ra05600j (PMC9042239; doi:10.1039/d1ra05600j)
Supplement: RA-011-D1RA05600J-s001 [file RA-011-D1RA05600J-s001.pdf]

Electronic Supplementary Material (ESI) for RSC Advances.
This journal is © The Royal Society of Chemistry 2021

ESI: Systematically investigating CO_2 : NH_3 ice mixtures using mid-IR and VUV spectroscopy - Part 2: electron and thermal processing

Rachel L. James, Sergio Ioppolo, Søren V. Hoffmann, Nykola C. Jones, Nigel J. Mason and Anita Dawes

S1 Experimental

Table S1 The film thickness of samples used and normalisation factor applied in the presented experiments. See text for details. Mid-IR spectra were normalised to a thickness of 300 nm and VUV spectra were normalised to a thickness of 200 nm.

Spectroscopic study	Ratio	Sample thickness (nm)	Normalisation factor
Mid-IR	1:0	475	0.84
	4:1	382	1.05
	2:1	424	0.94
	1:1	398	1.01
	1:2	470	0.85
	1:5	476	0.84
	1:10	455	0.88
	0:1	418	0.96
VUV	1:0	284	0.70
	4:1	237	0.84
	2:1	227	0.88
	1:3	154	1.30
	0:1	119	1.68

S1.1 Electron penetration depth: CASINO user inputs

Table S2 gives the weighted density of the CO_2:NH_3 mixtures input into CASINO and the corresponding estimated penetration depths. A weighted density was calculated for mixtures of CO_2:NH_3 ices and was dependent on the fractional proportion of NH_3 (a) and the fractional proportion of CO_2 (b) of the stoichiometric mixture:

$$\rho_m = (a\times\rho_{NH3})+(b\times\rho_{CO2}) \quad (1)$$

where ρ_{NH3} is the density of pure NH_3 ice (0.74 g cm^{-3})[1] and ρ_{CO2} is the density of pure CO_2 ice (1.11 g cm^{-3}).[1]

Table S2 CASINO estimated 1 keV electron penetration depth.

Spectroscopic study	Ratio	Density (g cm^{-3})	Penetration depth (nm)
Mid-IR	1:0	1.11	61
	4:1	1.06	54
	2:1	1.02	64
	1:1	0.98	57
	1:2	0.93	78
	1:5	0.89	74
	1:10	0.86	64
	0:1	0.74	84
VUV	1:0	1.11	60
	4:1	1.06	55
	2:1	1.02	68
	1:3	0.91	64
	0:1	0.74	65

S2 Additional mid-IR spectra

S2.1 Deposition at 20 K

Table S3 Band assignments and positions of the vibrational modes of pure CO_2, pure NH_3 and unirradiated CO_2:NH_3 mixtures deposited at 20 K before irradiation with 1 keV electrons.

Molecule	Vib. Mode	Assignment	Position (cm^{-1})								
			Ref.	1:0	4:1	2:1	1:1	1:2	1:5	1:10	0:1
CO_2	v_1+v_3	combination	2	3709	3702	3701	3701	3700	3698	3698	
	$2v_2+v_3$	combination	2	3601	3596	3595	3594	3593	3592	3591	
	v_3	C–O asym. stretch (LO)	3	2380	2374	2369	2367	2360	2362		
	v_3	C–O asym. stretch (TO)	2,3	2345	2335	2335	2336	2338	2342	2343	
$^{13}CO_2$	v_3	asym. Stretch	2	2283	2280	2279	2278	2278	2277	2277	
CO_2:NH_3 complex			4		3418	3414	3413	3405			
			4		3247	3249	3249	3248	3248		
NH_3	v_3	N–H asym. Stretch	5		3387	3382	3381	3376	3373	3373	3377
	$2v_4$	overtone	5		3313	3313	3308	3310	3304	3292	3294
	v_1	N–H sym. Stretch	5		3222	3221	3220	3218	3216	3215	3213
	v_4+v_L	combination	5						1860	1861	1883
	v_4	deformation	5		1631	1630	1630	1627	1627	1626	1626
	v_2	umbrella	5		1062	1068	1067	1067	1072	1072	1073

S2.1 Deposition at 20 K

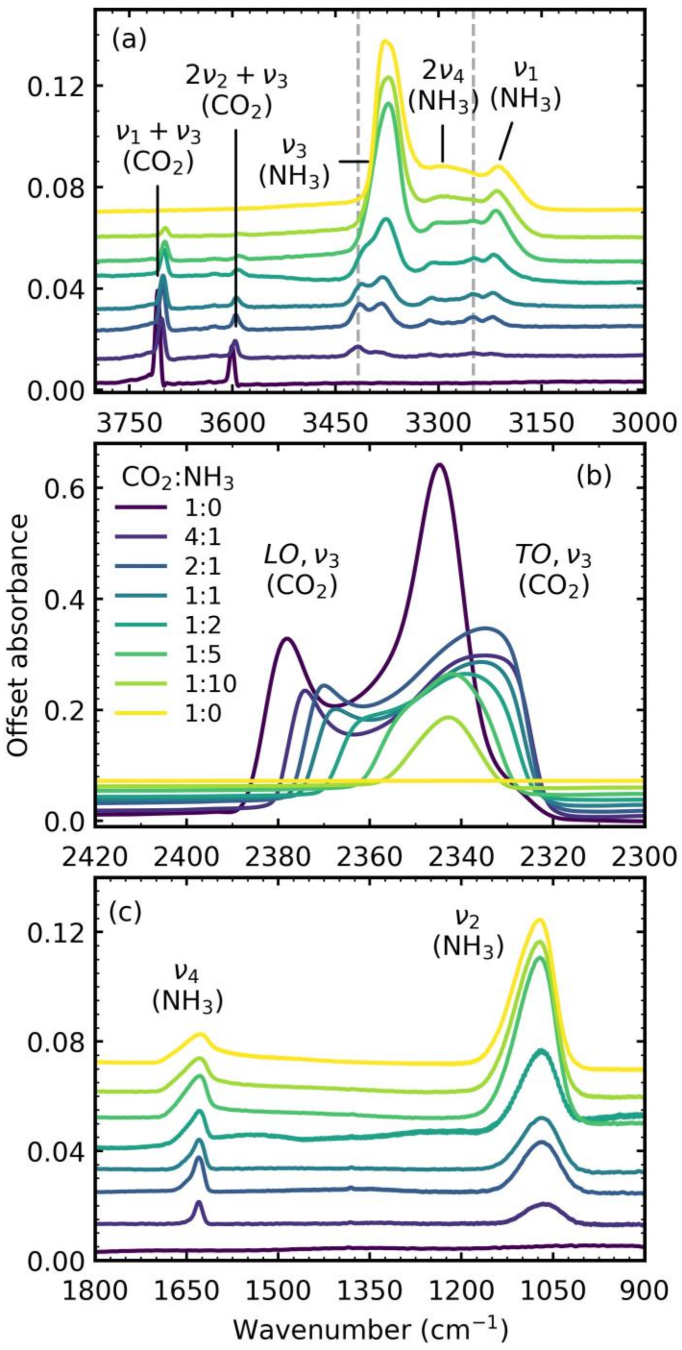

Figure S1 Mid-IR spectra of CO_2:NH_3 mixtures (4:1, 2:1, 1:1, 1:2, 1:5 & 1:10) deposited at 20 K compared with pure CO_2 (1:0) and pure NH_3 (0:1). (**a)** Dashed lines represent CO_2:NH_3 molecular complex vibrational features. (**b)** LO-TO splitting of the ν_3 fundamental mode of CO_2. (**c)** NH_3 ν_4 and ν_2 modes. Band assignments are given in Table S3. Spectra are offset on the y-axis for clarity and normalised to a thickness of 300 nm.

S2.2 Electron processing at 20 K

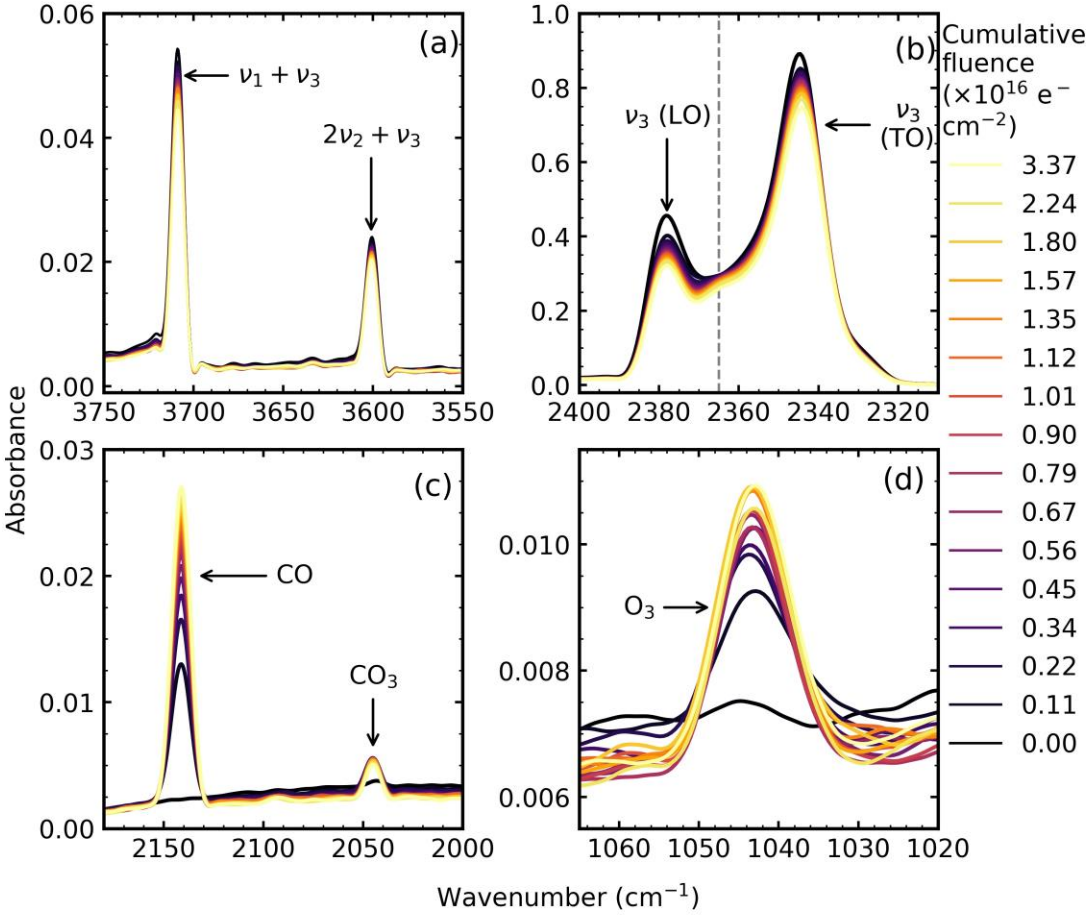

Figure S2 Mid-IR spectra of pure CO_2 deposited at 20 K and then processed with 1 keV electrons at discrete intervals to a total fluence of 3.37×10^{16} e$^-$ cm^{-2}. **(a)** CO_2 combination modes; **(b)** LO-TO splitting of the ν_3 mode; (c) Formation of CO and CO_3^-; **(d)** Formation of O_3.

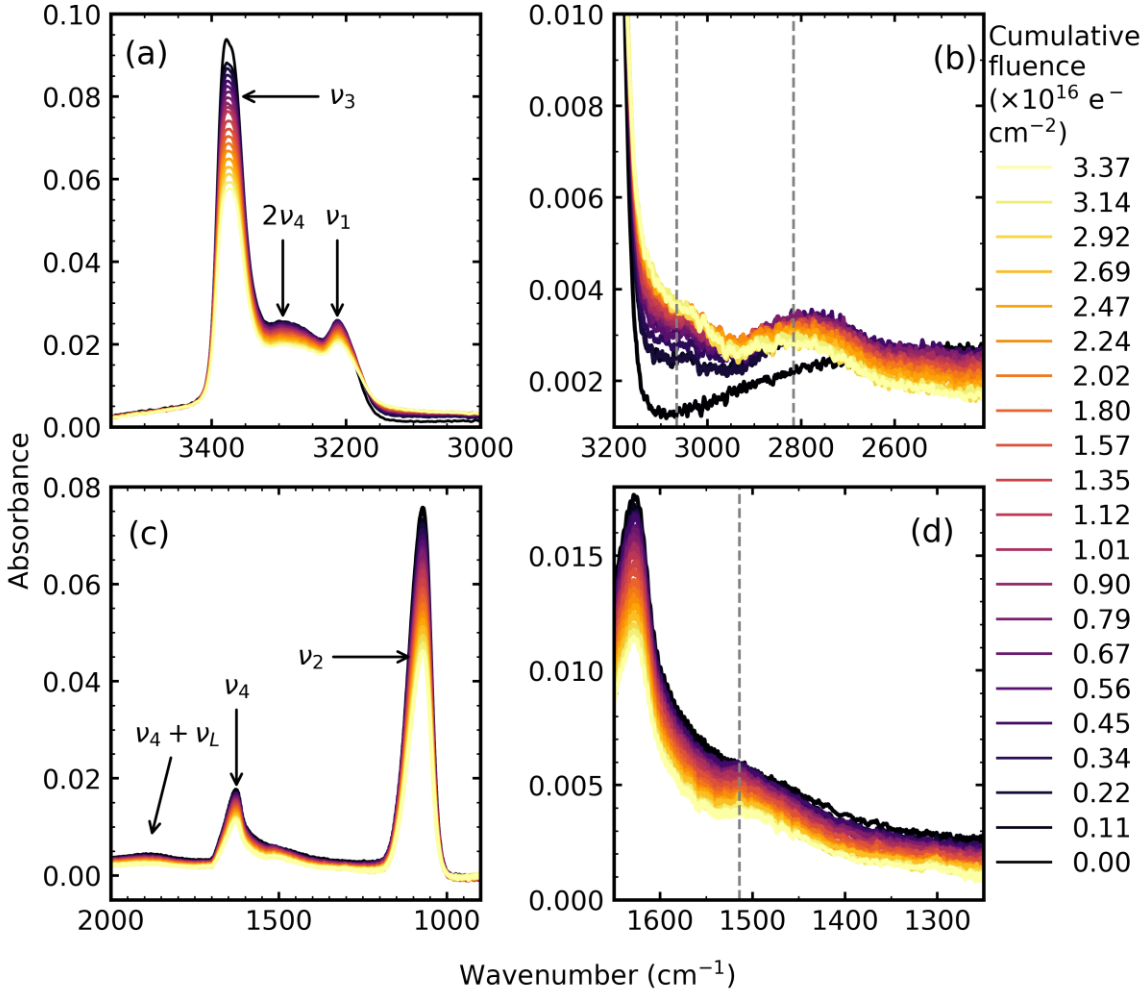

Figure S3 Mid-IR spectra of pure NH_3 deposited at 20 K and then processed with 1 keV electrons at discrete intervals to a total fluence 3.37×10^{16} e^{-} cm^{-2}. **(a)** N−H stretching region; (**b)** New features due to electron processing; **(c)** $\nu_4 + \nu_L$, ν_4 and ν_2 modes; **(d)** New feature due to electron processing.

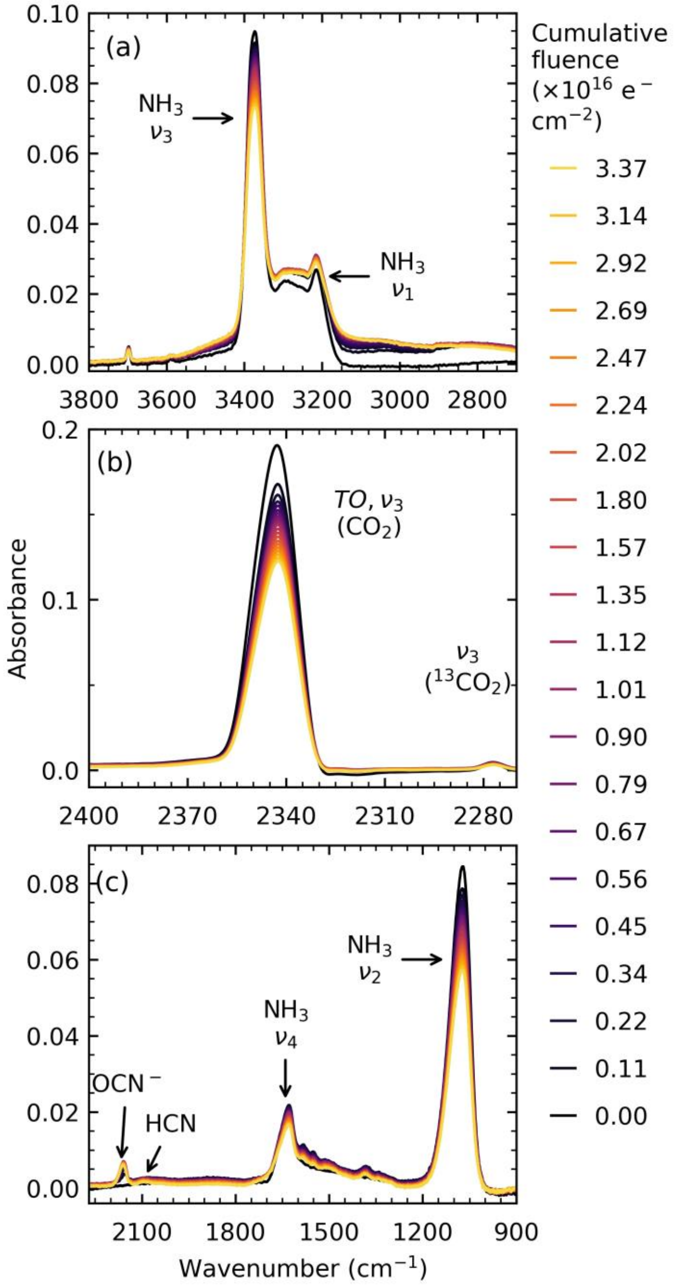

Figure S4 Example mid-IR spectra of a CO_2:NH_3 mixture in a 1:10 ratio irradiated with 1 keV electron at 20 K to a total fluence of 3.37×10^{16} e^{-} cm^{-2}. **(a)** O−H/N−H stretching region between 3500–2900 cm^{-1}. **(b)** No observed LO-TO splitting of the ν_3 vibrational mode of CO_2. **(c)** Several new features formed including OCN^{-} & HCN and new absorptions between 1750–250 cm^{-1}.

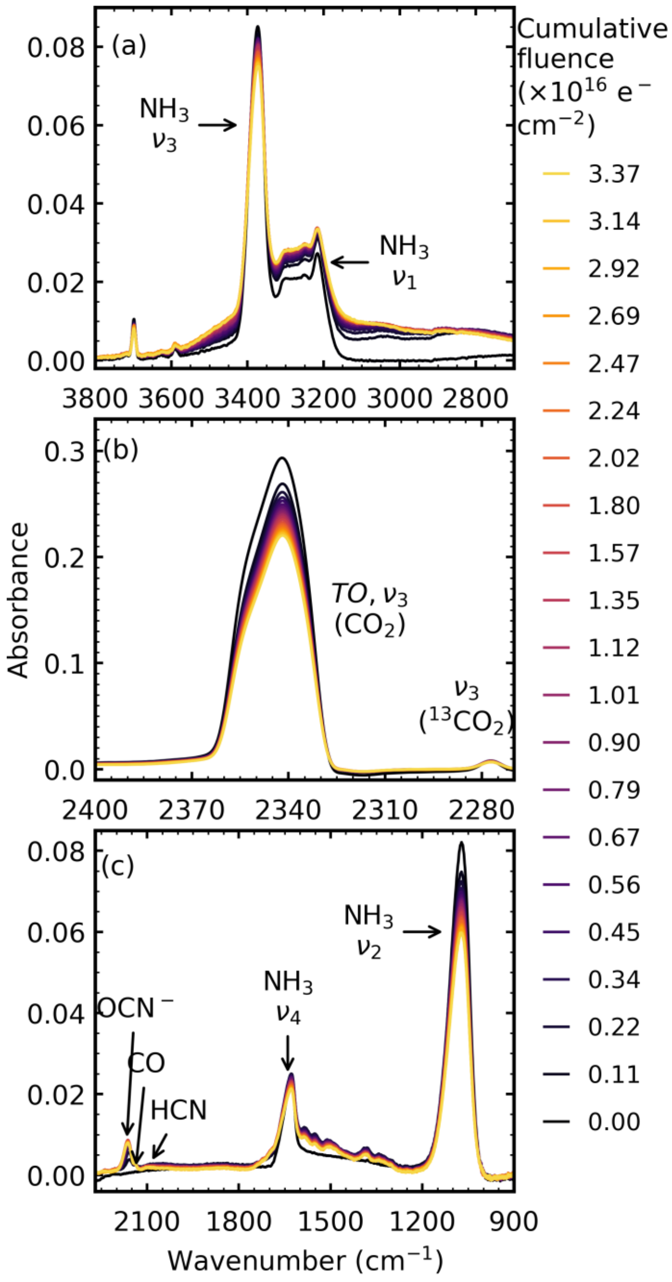

Figure S5 Example mid-IR spectra of a CO_2:NH_3 mixture in a 1:5 ratio irradiated with 1 keV electron at 20 K to a total fluence of 3.37×10^{16} e^- cm^{-2}. **(a)** O−H/N−H stretching region between 3500–2900 cm^{-1}; **(b)** LO-TO splitting of the ν_3 vibrational mode of CO_2; **(c)** Several new features formed including OCN^-, CO & HCN and absorptions between 1750–1250 cm^{-1}.

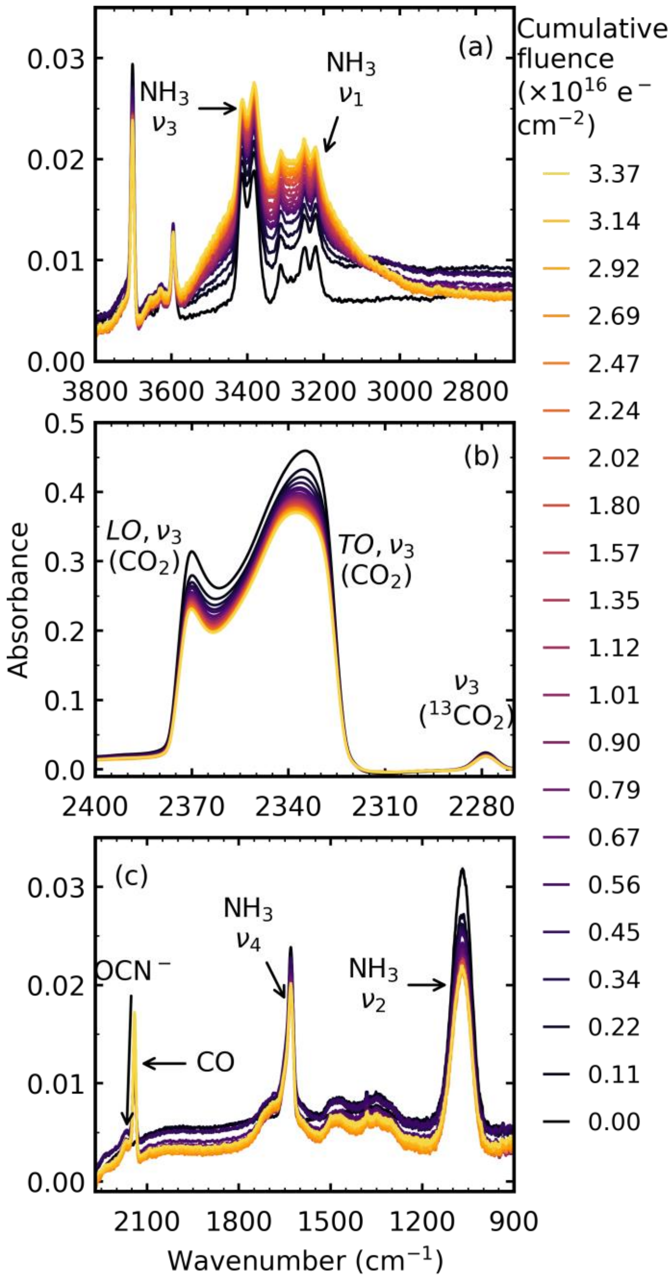

Figure S6 Example mid-IR spectra of a CO_2:NH_3 mixture in a 2:1 ratio irradiated with 1 keV electron at 20 K to a total fluence of 3.37×10^{16} e^- cm^{-2}. **(a)** O−H/N−H stretching region between 3500–2900 cm^{-1}; **(b)** LO-TO splitting of the ν_3 vibrational mode of CO_2; **(c)** Several new features formed including OCN^-, CO & HCN and absorptions between 1750–1250 cm^{-1}.

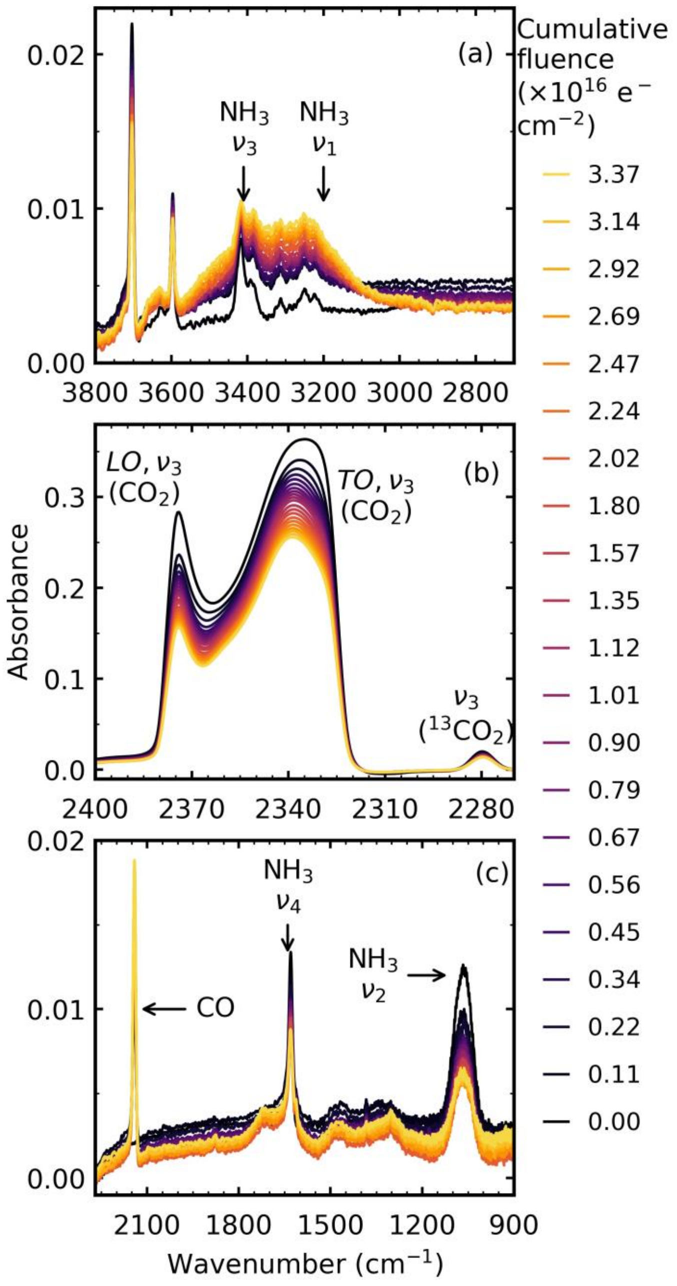

Figure S7 Example mid-IR spectra of a CO_2:NH_3 mixture in a 4:1 ratio irradiated with 1 keV electron at 20 K to a total fluence of 3.37×10^{16} e^- cm^{-2}. **(a)** O−H/N−H stretching region between 3500–2900 cm^{-1}; **(b)** LO-TO splitting of the ν_3 vibrational mode of CO_2; **(c)** Several new features formed including OCN^-, CO & HCN and absorptions between 1750–1250 cm^{-1}.

S2.3 Thermal processing

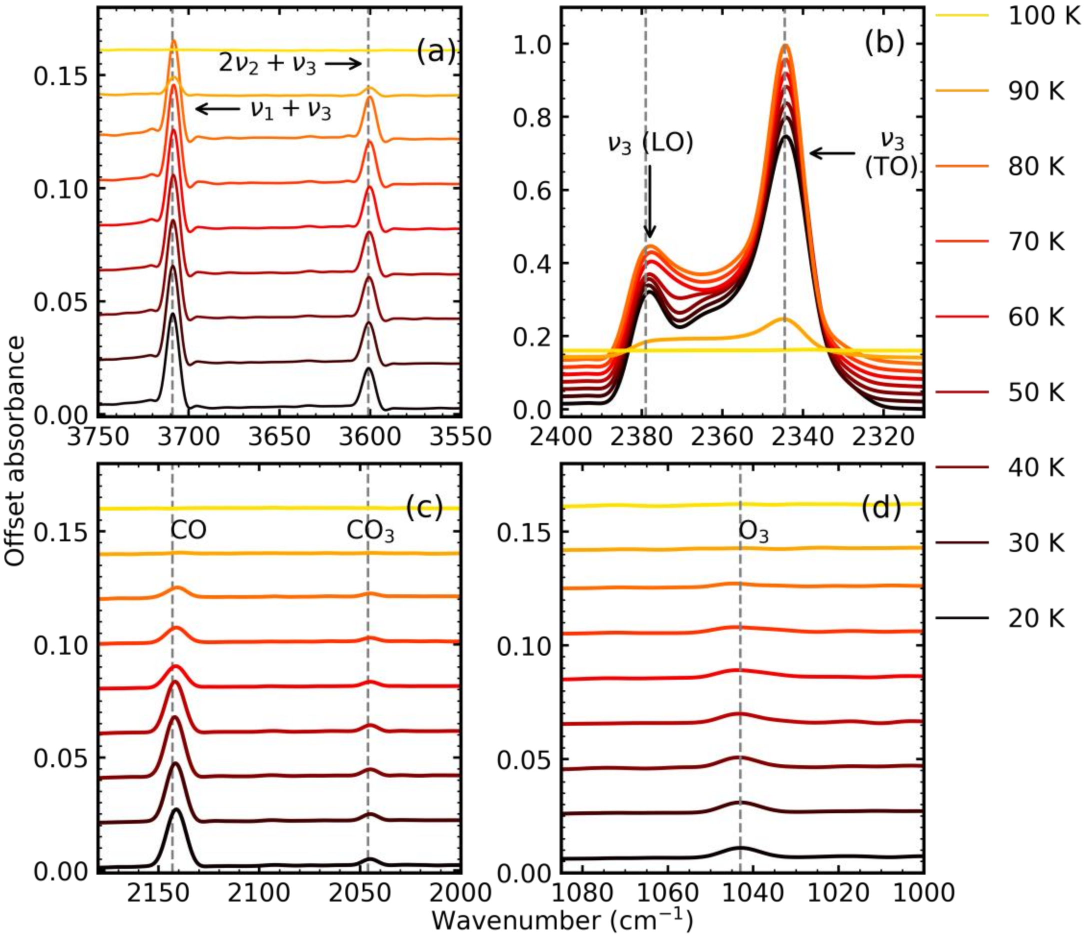

Figure S8 Mid-IR spectra of the thermal processing of pure CO_2 after deposition at 20 K and irradiation with 1 keV electrons to a fluence of 3.37×10^{16} e^{-} cm^{-2} at 20 K. Spectra are offset on the y-axis for clarity. **(a)** CO_2 combination modes; **(b)** LO-TO splitting of the ν_3 mode; **(c)** CO and CO_3^{-}; **(d)** O_3.

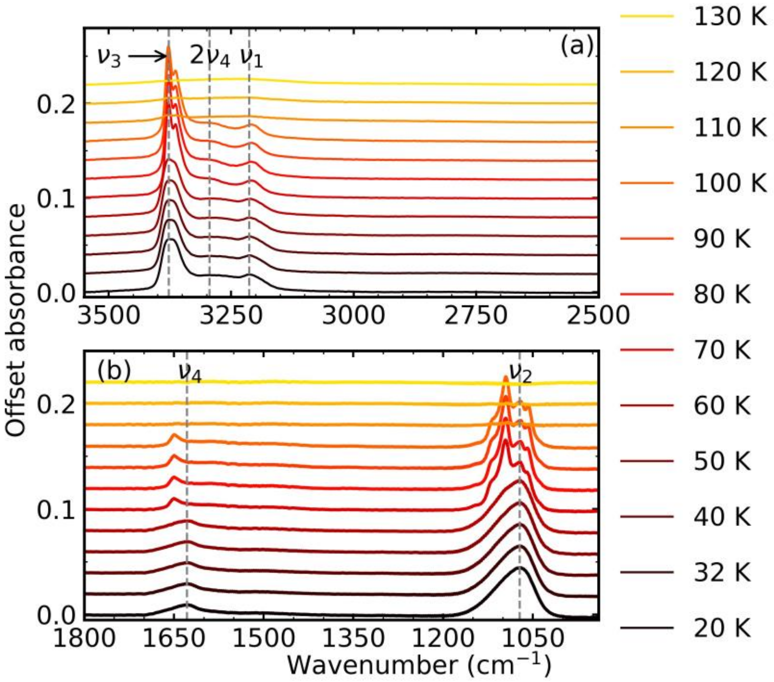

Figure S9 Mid-IR spectra of the thermal processing of pure NH_3 after deposition at 20 K and irradiation with 1 keV electrons to a fluence of 3.37×10^{16} e^{-} cm^{-2} at 20 K. Spectra are offset on the y-axis for clarity. **(a)** N−H stretching region; **(b)** ν_4 and ν_2 modes.

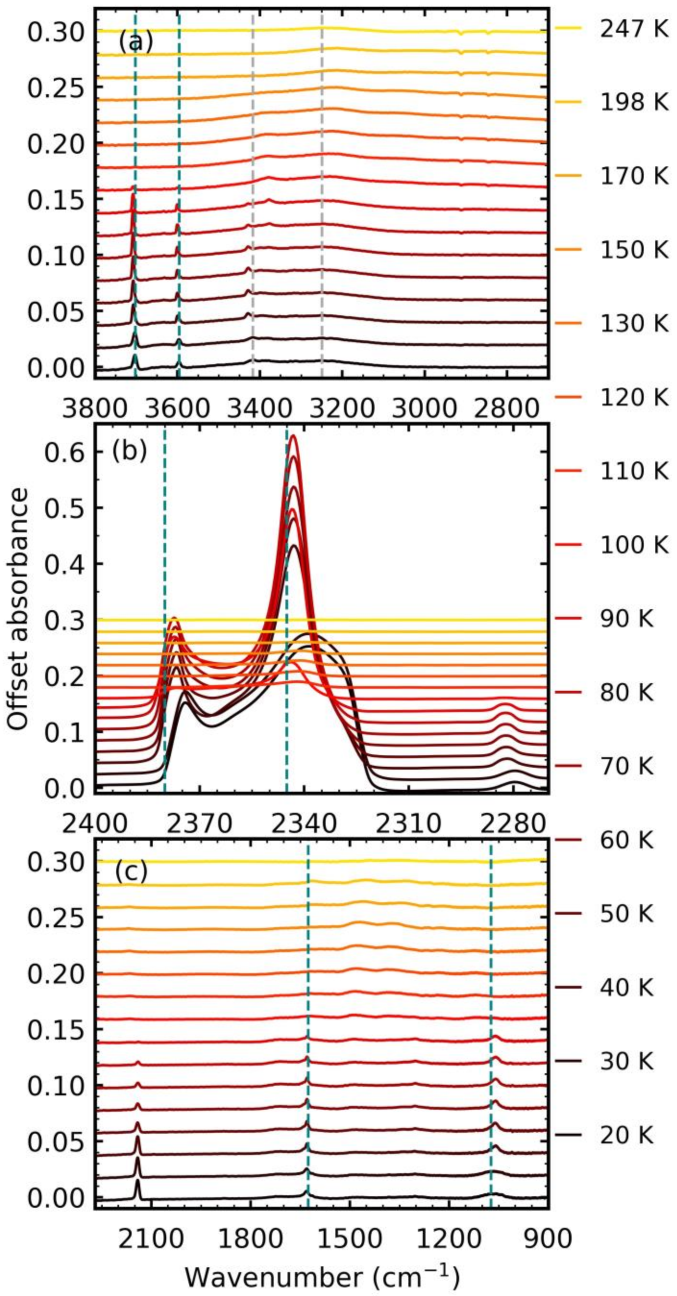

Figure S10 Mid-IR spectra of the thermal processing results of a CO_2:NH_3 mixture in a 4:1 ratio after deposition and irradiation with 1 keV electrons to a fluence of 3.37×10^{16} e^- cm^{-2} at 20 K. Spectra are offset on the y-axis for clarity. **(a)** Segregation of the mixture was observed through the shift in position of the CO_2 vibrational modes towards pure CO_2 position which are indicated by blue dashed lines. Grey dashed lines indicate CO_2:NH_3 molecular complex vibrational modes which disappeared between 60–70 K. **(b)** LO-TO splitting of the v_3 vibrational mode of CO_2. **(c)** Thermally induced reaction at 80 K.

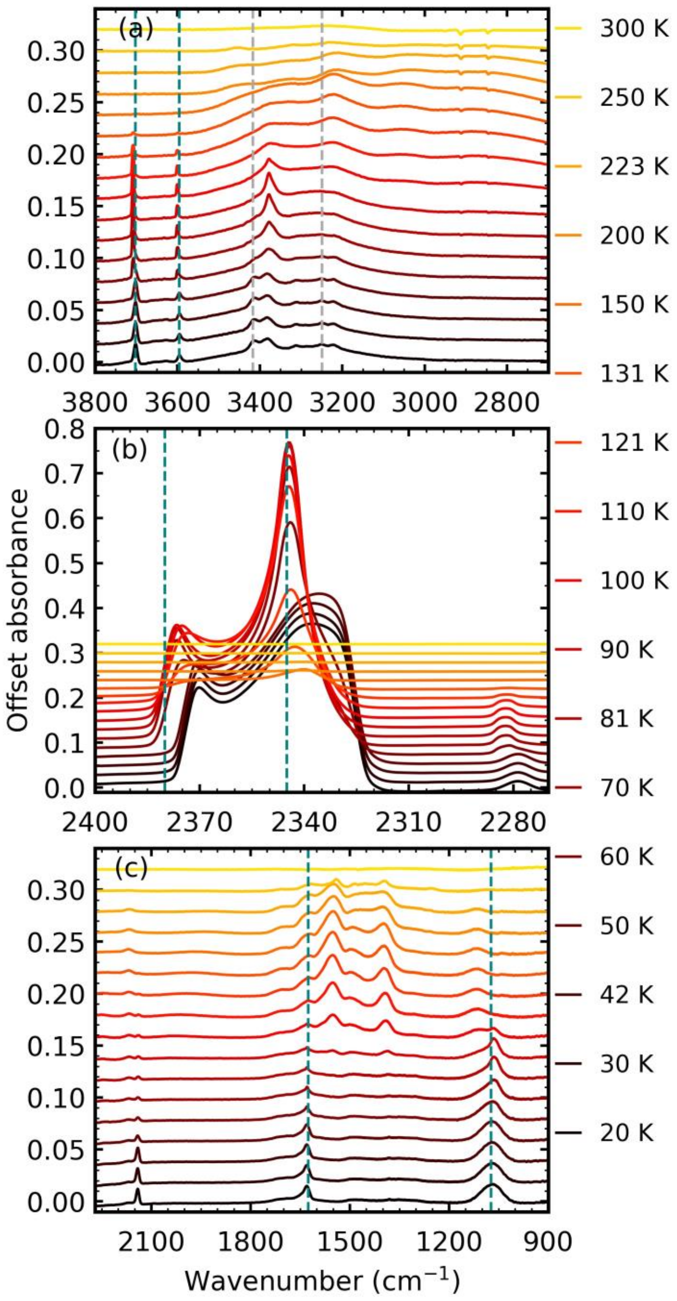

Figure S11 Mid-IR spectra of the thermal processing results of a CO_2:NH_3 mixture in a 2:1 ratio after deposition and irradiation with 1 keV electrons to a fluence of $3.37{\times}10^{16}$ e^- cm^{-2} at 20 K. Spectra are offset on the y-axis for clarity. **(a)** Segregation of the mixture was observed through the shift in position of the CO_2 vibrational modes towards pure CO_2 position which are indicated by blue dashed lines. Grey dashed lines indicate CO_2:NH_3 molecular complex vibrational modes which disappeared between 60–70 K. **(b)** LO-TO splitting of the v_3 vibrational mode of CO_2. **(c)** Thermally induced reaction at 80 K.

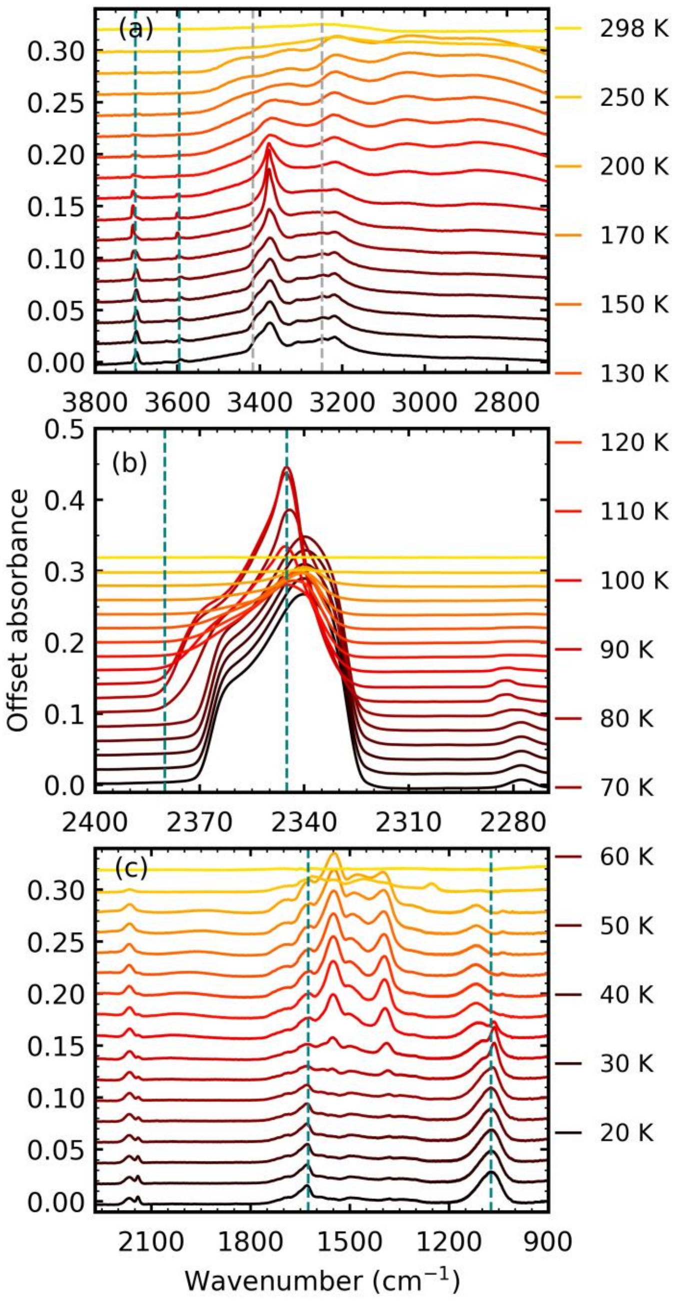

Figure S12 Mid-IR spectra of the thermal processing results of a CO_2:NH_3 mixture in a 1:2 ratio after deposition and irradiation with 1 keV electrons to a fluence of 3.37×10^{16} e^- cm^{-2} at 20 K. Spectra are offset on the y-axis for clarity. **(a)** Segregation of the mixture was observed through the shift in position of the CO_2 vibrational modes towards pure CO_2 position which are indicated by blue dashed lines. Grey dashed lines indicate CO_2:NH_3 molecular complex vibrational modes which disappeared between 60–70 K. **(b)** LO-TO splitting of the ν_3 vibrational mode of CO_2. **(c)** Thermally induced reaction at 80 K.

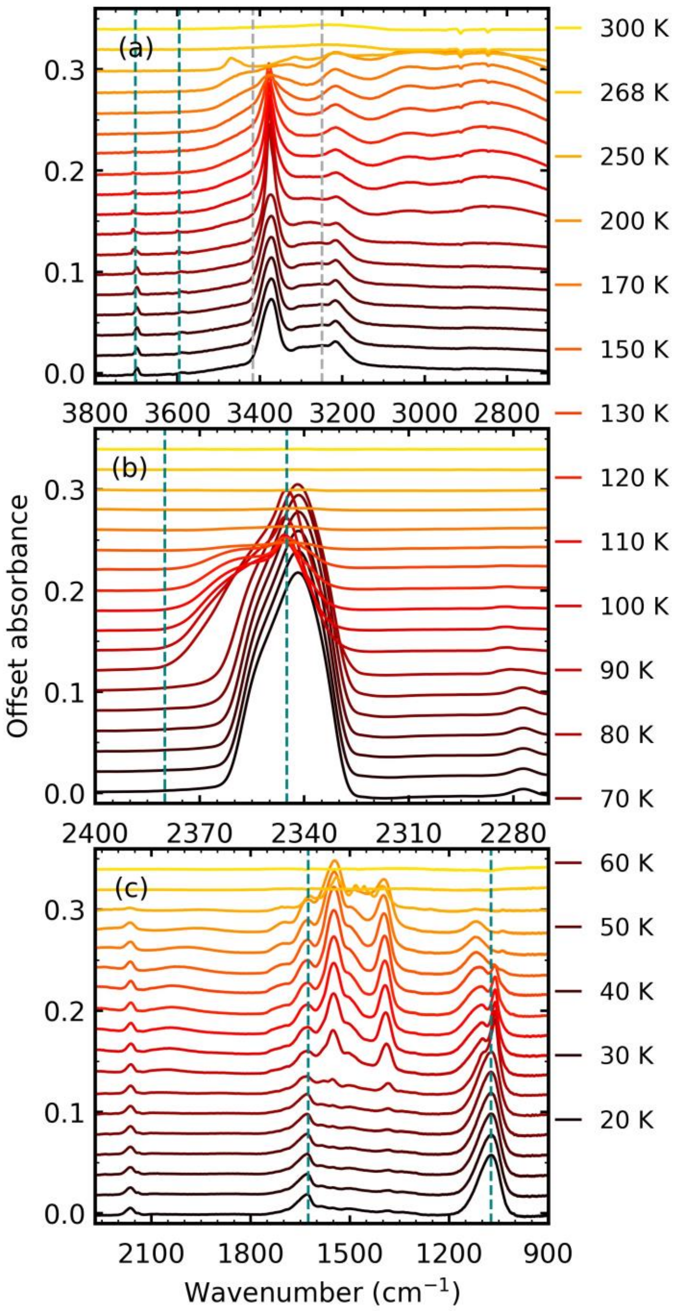

Figure S13 Mid-IR spectra of the thermal processing results of a CO_2:NH_3 mixture in a 1:5 ratio after deposition and irradiation with 1 keV electrons to a fluence of 3.37×10^{16} $e^- cm^{-2}$ at 20 K. Spectra are offset on the y-axis for clarity. **(a)** Segregation of the mixture was observed through the shift in position of the CO_2 vibrational modes towards pure CO_2 position which are indicated by blue dashed lines. Grey dashed lines indicate CO_2:NH_3 molecular complex vibrational modes which disappeared between 60–70 K. **(b)** LO-TO splitting of the ν_3 vibrational mode of CO_2. **(c)** Thermally induced reaction at 80 K.

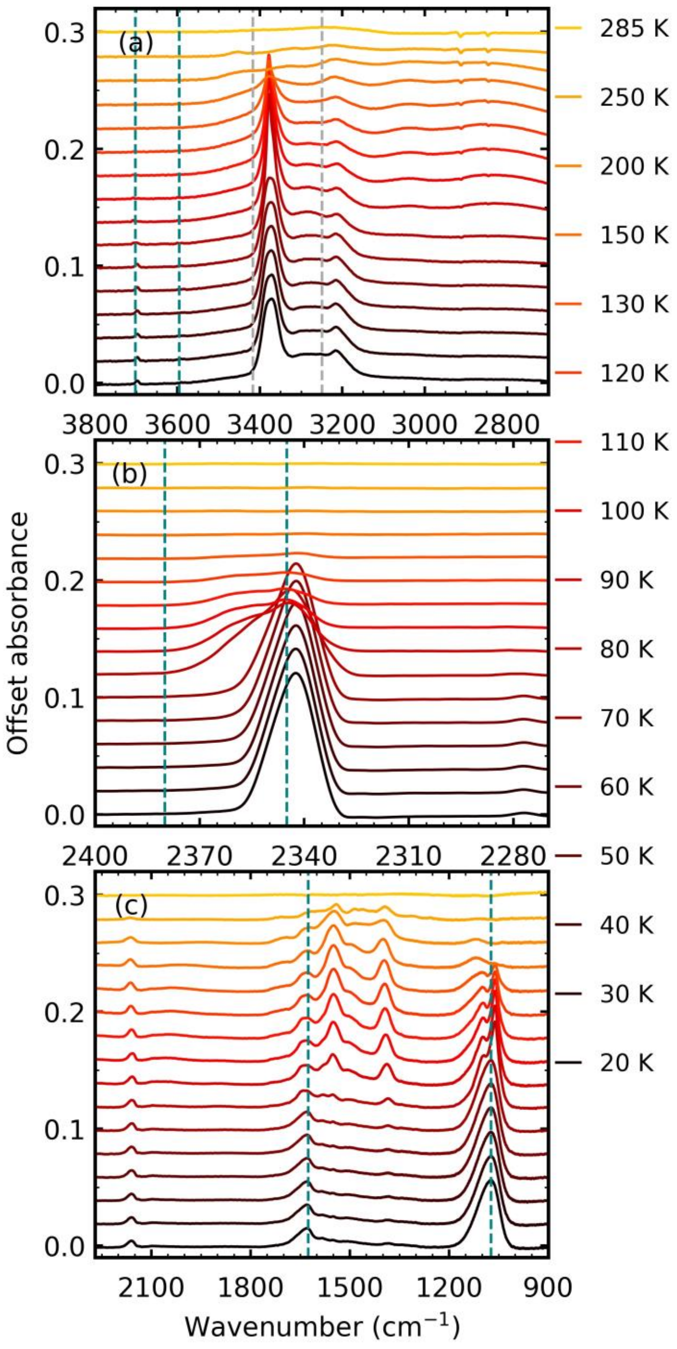

Figure S14 Mid-IR spectra of the thermal processing results of a CO_2:NH_3 mixture in a 1:10 ratio after deposition and irradiation with 1 keV electrons to a fluence of 3.37×10^{16} e^{-} cm^{-2} at 20 K. Spectra are offset on the y-axis for clarity. **(a)** Segregation of the mixture was observed through the shift in position of the CO_2 vibrational modes towards pure CO_2 position which are indicated by blue dashed lines. Grey dashed lines indicate CO_2:NH_3 molecular complex vibrational modes which disappeared between 60–70 K. **(b)** LO-TO splitting of the v_3 vibrational mode of CO_2. **(c)** Thermally induced reaction at 80 K.

S3 Additional VUV spectra

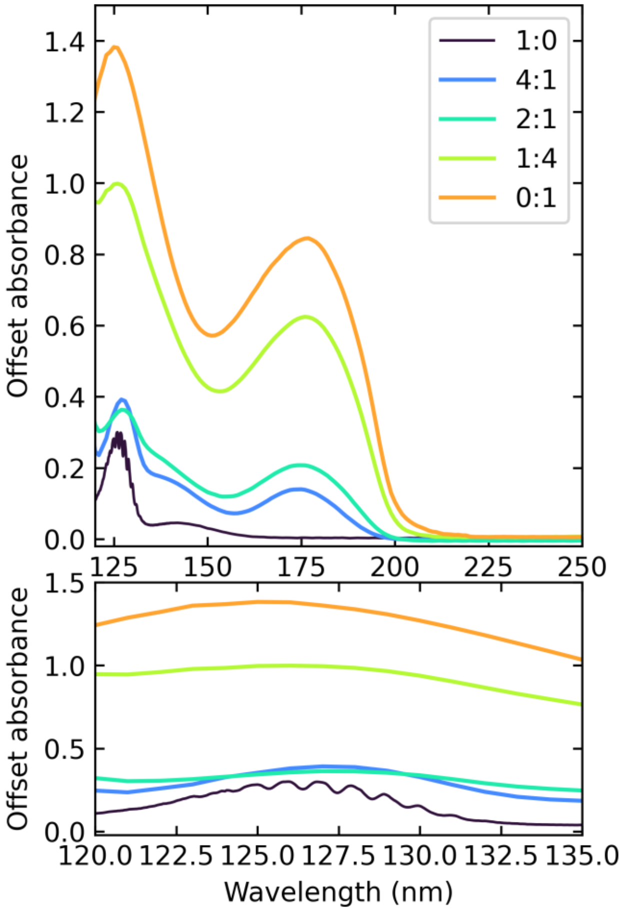

Figure S15 VUV spectra of CO_2:NH_3 mixtures (4:1, 2:1 & 1:3) compared with pure CO_2 (1:0) and pure NH_3 (0:1) between 120–340 nm (top plot). Bottom plot shows a close up of the region between 120–135 nm. Normalised to a thickness of 200 nm.

Notes and references

[1] M. Bouilloud, N. Fray, Y. Bénilan, H. Cottin, M.-C. Gazeau and A. Jolly, *Mon. Not. R. Astron. Soc.*, 2015, 451, 2145–2160.
[2] P. A. Gerakines, W. A. Schutte, J. M. Greenberg and E. F. van Dishoeck, *Astron. Astrophys*, 1995, 296, 810.
[3] G. A. Baratta and M. E. Palumbo, *J. Opt. Soc. Am. A*, 1998, 15, 3076–3085.
[4] J. B. Bossa, F. Duvernay, P. Theulé, F. Borget and T. Chiavassa, *Chem. Phys.*, 2008, 354, 211–217.
[5] F. P. Reding and D. F. Hornig, *J. Chem. Phys.*, 1951, 19, 594–601.
